# Systematic review of knowledge translation strategies in the allied health professions

**DOI:** 10.1186/1748-5908-7-70

**Published:** 2012-07-25

**Authors:** Shannon D Scott, Lauren Albrecht, Kathy O’Leary, Geoff DC Ball, Lisa Hartling, Anne Hofmeyer, C Allyson Jones, Terry P Klassen, Katharina Kovacs Burns, Amanda S Newton, David Thompson, Donna M Dryden

**Affiliations:** 1 Faculty of Nursing, University of Alberta, Level 3, Edmonton Clinic Health Academy, Edmonton, AB, Canada; 2 Department of Pediatrics, Faculty of Medicine and Dentistry, University of Alberta, 8213 Aberhart Centre, Edmonton, AB, Canada; 3 Alberta Research Centre for Health Evidence, University of Alberta, Level 4, Edmonton Clinic Health Academy, Edmonton, AB, Canada; 4 School of Nursing and Midwifery, University of South Australia, Adelaide, South Australia, Australia; 5 Department of Physical Therapy, Faculty of Rehabilitation Medicine, University of Alberta, 3-48 Corbett Hall, Edmonton, AB, Canada; 6 Manitoba Institute of Child Health, Department of Pediatrics and Child Health, University of Manitoba, Level 5, John Buhler Research Centre, Winnipeg, MB, Canada; 7 Winnipeg Regional Health Authority, 650 Main Street, Winnipeg, MB, Canada; 8 Health Sciences Council and Interdisciplinary Health Research Academy, University of Alberta, 3–398 Edmonton Clinic Health Academy, Edmonton, AB, Canada; 9 Glenrose Rehabilitation Hospital, 10230 111 Avenue, Edmonton, AB, Canada; 10 Women and Children’s Health Research Institute, University of Alberta, 4–081 Edmonton Clinic Health Academy, Edmonton, AB, Canada; 11 Stollery Children’s Hospital, 8440 112 Street, Edmonton, AB, Canada; 12 Northern Ontario School of Medicine, 955 Oliver Road, Thunder Bay, ON, Canada

**Keywords:** Allied health, Knowledge translation, Research use, Behaviour change interventions, Systematic review

## Abstract

**Background:**

Knowledge translation (KT) aims to close the research-practice gap in order to realize and maximize the benefits of research within the practice setting. Previous studies have investigated KT strategies in nursing and medicine; however, the present study is the first systematic review of the effectiveness of a variety of KT interventions in five allied health disciplines: dietetics, occupational therapy, pharmacy, physiotherapy, and speech-language pathology.

**Methods:**

A health research librarian developed and implemented search strategies in eight electronic databases (MEDLINE, CINAHL, ERIC, PASCAL, EMBASE, IPA, Scopus, CENTRAL) using language (English) and date restrictions (1985 to March 2010). Other relevant sources were manually searched. Two reviewers independently screened the titles and abstracts, reviewed full-text articles, performed data extraction, and performed quality assessment. Within each profession, evidence tables were created, grouping and analyzing data by research design, KT strategy, targeted behaviour, and primary outcome. The published descriptions of the KT interventions were compared to the Workgroup for Intervention Development and Evaluation Research (WIDER) Recommendations to Improve the Reporting of the Content of Behaviour Change Interventions.

**Results:**

A total of 2,638 articles were located and the titles and abstracts were screened. Of those, 1,172 full-text articles were reviewed and subsequently 32 studies were included in the systematic review. A variety of single (n = 15) and multiple (n = 17) KT interventions were identified, with educational meetings being the predominant KT strategy (n = 11). The majority of primary outcomes were identified as professional/process outcomes (n = 25); however, patient outcomes (n = 4), economic outcomes (n = 2), and multiple primary outcomes (n = 1) were also represented. Generally, the studies were of low methodological quality. Outcome reporting bias was common and precluded clear determination of intervention effectiveness. In the majority of studies, the interventions demonstrated mixed effects on primary outcomes, and only four studies demonstrated statistically significant, positive effects on primary outcomes. None of the studies satisfied the four WIDER Recommendations.

**Conclusions:**

Across five allied health professions, equivocal results, low methodological quality, and outcome reporting bias limited our ability to recommend one KT strategy over another. Further research employing the WIDER Recommendations is needed to inform the development and implementation of effective KT interventions in allied health.

## Background

Strategies to close the gap between research and practice, in the context of the knowledge user, have been identified as a means to realize and maximize the benefits of research through improved health outcomes, better health services and products, strengthened healthcare systems, and more effective health service delivery [1-3]. The field of knowledge translation (KT) aims to close the research-practice gap through the development and implementation of KT strategies, which include a variety of professional, financial, organizational, and regulatory interventions aimed at changing healthcare professional behaviour (*i.e.*, change decision making, change treatment, and management) to be aligned with evidence-based recommendations.

While there is a growing understanding of the implementation and effectiveness of KT strategies, the bulk of the research evidence comes from the medical *e.g.*, [4-9] and nursing literature *e.g.*, [10,11]. Presently, evidence supporting the need for interdisciplinary collaboration within healthcare is mounting both nationally [12-16] and internationally [17]. As interprofessional collaboration and evidence-based practice become increasingly important to effective health delivery, a better understanding of how to increase research use by all health professions is critical. Currently, we have limited knowledge of the use of KT strategies in allied health professions.

Presently, the allied health KT literature contains two systematic reviews that have examined strategies to aide guideline implementation [18,19], which is one of many ways to put research into practice. In the review by Thomas *et al*. [18], only one of 18 studies focused on dietitians (*i.e.*, allied health professionals), while the remaining seventeen studies targeted nurses and physicians. Thus, limited conclusions could be drawn in relation to allied health professional practice. In the second systematic review, Hakkennes and Dodd [19] examined 14 studies that used single and multiple KT interventions. Education-related interventions were the most commonly used interventions in their review; however, Hakkennes and Dodd [19] described equivocal findings of the guideline implementation strategies across studies.

In addition to the aforementioned systematic reviews on guideline implementation, a third systematic review by Menon *et al*. [20] examined a variety of single and multi-component KT strategies to improve knowledge and attitudes regarding evidence-based practice and evidence-based practice behaviours in occupational therapy and physiotherapy practice. Overall, the findings highlighted that there was limited evidence for the effectiveness of the KT strategies; however, they suggested strong evidence pointed to the effectiveness of an active, multi-component KT intervention for changing practice behaviours of physiotherapists (*i.e.*, physical therapist).

Broadly, there has been limited success identifying consistently effective KT interventions. Partially, this may be attributed to a general lack of theory-driven KT interventions [21]. Recent evidence suggests that less than 10% of studies on guideline implementation explicitly reported a theoretical rationale for the selected KT intervention [22]. Theory-driven interventions use established theory to select and develop the KT interventions through articulating the desired behaviour change, as well as the factors and mechanisms that may shape this change, such as autonomy and scope of practice (which may vary widely across health disciplines, and potentially across geographical jurisdictions) [23-27]. A theoretical-informed approach offers the advantage of a generalizable framework to: inform the development and delivery of interventions; guide evaluation; explore moderating factors and causal mechanisms; and facilitate a better understanding of the generalizability and replicability of implementation interventions. While there is growing interest in the use of theory-informed knowledge translation interventions [28,29], little empirical evidence exists that interventions designed using theory are superior to non-theory informed interventions [30], however evidence is starting to emerge [31]. As a result, the generalization of research evidence about KT interventions across disciplines is questionable given the lack of explicit theory-informed interventions. Systematic reviews must reflect these important discipline-specific nuances through synthesizing knowledge from disciplines that have similar scope of practice and autonomy, for example. A related point that is commonly overlooked in the literature, yet bears important implications for KT, is that changes in knowledge and attitudes do not necessarily facilitate behaviour change (*i.e.*, increased use of evidence-based practices) [32].

Many professions can be considered part of the allied health sector. For the purposes of this project, we conceptualized allied health professionals to encompass and reflect five key health professions allied to medicine and nursing in the Canadian acute care context. These professions include dietetics, pharmacy, and rehabilitation medicine (*i.e.*, physiotherapy, occupational therapy, speech-language pathology). All full rationale for the selection of these five professions under the umbrella term allied health professionals is described in the study protocol [33]. Because allied health is uniquely positioned in the Canadian healthcare landscape, this review is intended to broadly examine the field of allied health. However, it is also important to recognize that the nature and structure of the work differs within these disciplines; thus, this review also examined each of these professions individually in order to identify important similarities and differences. The objective of this project was to:

1. systematically locate, assess, and report on studies from each respective allied health profession [1] that have investigated the effects of KT interventions;

2. evaluate the interventions used to translate research into practice in terms of changes at the healthcare system, health provider, and/or patient level;

3. describe how the interventions worked and the modifying variables relevant to the respective context (*i.e.*, for whom does the intervention work, under what circumstances, and in what manner) [34];

4. provide possible strategies to facilitate KT for allied healthcare professionals and decision makers responsible for policy and institution/unit protocols in healthcare settings;

5. offer guidance for KT researchers in terms of the development of KT interventions for interprofessional healthcare teams.

## Methods

This review followed a modified systematic review protocol in order to synthesize diverse forms of research evidence [35]. The study procedures we applied are documented in a previous publication [33]. Our methodological approach for extracting data, assessing intervention reporting, and conducting methodological quality assessment of the qualitative studies are described in detail below because they were either not described in the study protocol or they were adjusted during the systematic review process.

### Literature search

A health research librarian developed and implemented search strategies in eight electronic databases (MEDLINE, CINAHL, ERIC, PASCAL, EMBASE, IPA, Scopus, CENTRAL) using language (English) and date restrictions (1985 to March 2010) (Additional File 1). The decision to restrict to English studies was informed by recent systematic research evidence that suggested there is no empirical evidence of bias if papers written in languages other than English (LOE) are excluded [36]. These date restrictions reflect the emergence of the evidence-based medicine/evidence-based practice and the knowledge translation movements and were purposively selected to capture all relevant literature. Relevant dissertations, reference lists of included studies, key journals and conference proceedings from 2005 to 2010 were also searched for relevant citations.

### Study inclusion criteria

Studies were included if they met the criteria outlined in Table 1. Studies were not excluded based upon research design.

**Table 1:** Inclusion criteria


**Study Design**	Primary research studies, including experimental, quasi-experimental, and non-experimental designs (*e.g.*, case study).
**Participants**	Dietitians, occupational therapists, pharmacists, physiotherapists, speech-language pathologists
**Interventions**	Interventions/strategies with a primary purpose of translating research (or enhancing research uptake) into clinical practice; examples of potential interventions include reminders, use of multidisciplinary teams, educational programs, researcher-clinician interventions.
**Outcomes**	Empirically assessed change (by way of quantitative or qualitative data) at the professional/process level (*e.g.*, change in clinical practice), patient level (*e.g.*, improved response to the clinical practice intervention) or the economic level (*e.g.*, change in costs).

### Study selection

Two reviewers independently screened the search results (*i.e.*, titles and abstracts) using broad criteria, and reviewed the full-text of potentially relevant articles using standard forms and predetermined inclusion criteria. Disagreements were resolved by discussion or third party adjudication.

### Data extraction

Study data were extracted using a modified version of the *Cochrane Effective Practice and Organisation of Care Review Group (EPOC) Data Collection Checklist* (Additional File 2) [37]. This classification scheme is currently used by the Cochrane Collaboration and widely used by other researchers. Research design was determined using an algorithm [38] (Additional File 3) because of its user-friendly format, which helped to identify a variety of research designs and standardize responses across reviewers. Other than to identify research design, the *EPOC Data Collection Checklist* was used as published. Data were extracted by one reviewer and verified by a second reviewer. Disagreements were resolved by discussion or third party adjudication.

### Intervention reporting

The descriptions of the KT interventions in each study were compared to the *Workgroup for Intervention Development and Evaluation Research (WIDER) Recommendations to Improve Reporting of the Content of Behaviour Change Interventions* (Additional File 4) [39]. These recommendations emerged from recommendations in the *Consolidated Standards of Reporting Trials (CONSORT)* agreement [40]. Developed in 2008, the WIDER Recommendations comprise four categories: detailed description of interventions in published papers; clarification of assumed change process and design principles; access to intervention manuals/protocols; and detailed description of active control conditions. In order for a description of a behaviour intervention to meet these criteria, the description must contain all of the components described within each recommendation. The descriptions of the KT interventions were compared to the WIDER Recommendations by one reviewer and verified by a second reviewer.

### Quality criteria

Two reviewers independently assessed the methodological quality of included studies; disagreements were resolved through discussion or third party adjudication. The methodological quality of quantitative studies was assessed using the *Quality Assessment Tool for Quantitative Studies*[41] (Additional File 5). The results from the tool led to an overall methodological rating of strong, moderate, or weak in eight sections, including: selection bias, study design, confounders, blinding, data collection methods, withdrawals/dropouts, intervention integrity, and analysis. This tool has been previously evaluated for content and construct validity, and inter-rater reliability, and meets accepted standards [42]. The methodological quality of qualitative studies was assessed using the *Quality Assessment Tool for Qualitative Studies* (Additional File 6) [43], which differs from the quality assessment tool described in the study protocol [33]. This framework assesses five aspects: the aims of the research; research methods and design; sampling; data collection and analysis; and results, discussion, and conclusions. Previous research on this tool has reported a kappa score of 0.526 [43], which indicates moderate inter-rater agreement [44]. We chose this tool because of structural similarities to the tool used for quantitative studies, which made the results more comparable, and the increased ease of presenting the individual quality criteria of the qualitative and quantitative studied included in this review in order to facilitate study comparison. All studies were included in data synthesis. No studies were excluded based on the quality assessment.

### Data analysis/synthesis

Several steps were taken to analyze and synthesize study data. First, analysis occurred on a profession-by-profession basis with study data grouped and analyzed by study design. Second, data were aggregated and analyzed according to the type of KT intervention strategies within each of the allied health professional disciplines. From this, we completed a descriptive (narrative) analysis of the included studies and identified potential patterns (*e.g.*, similarities, anomalies, *et al*.) in terms of targeted behaviours, study outcomes, and intervention effectiveness. This narrative review satisfied two goals: it allowed us to examine strategies that were successful across professions, and to explore what it was about different strategies that worked, for whom, and under what circumstances [34]. Third, we synthesized the evidence across the professions to reflect the interprofessional nature of Canada’s healthcare landscape. A detailed description of the planned meta-analysis is contained in the study protocol [33]; however, meta-analyses could not be conducted due to methodological and clinical heterogeneity of the studies.

## Results

**Figure 1:**
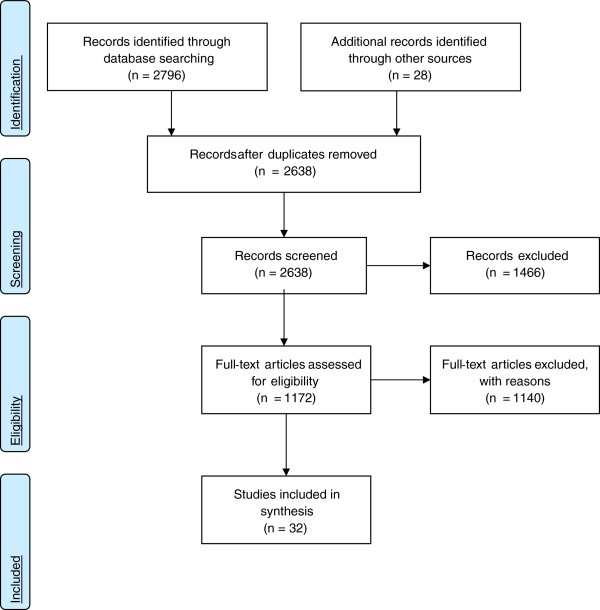
**PRISMA Flow Diagram**[77]**.**

Thirty-two studies met our inclusion criteria (Figure 1) [45-76]. Both quantitative (n = 29) and qualitative (n = 3) research designs were represented. Within each profession, the heterogeneity of the study designs, KT interventions, targeted behaviours, and study outcomes precluded combining comparable results. Table 2 summarizes the 32 studies, including important study elements, and is organized by discipline and study design.

### KT interventions

The 32 studies included diverse KT interventions. Fifteen studies investigated a single KT intervention (pharmacy n = 5, physiotherapy n = 2, occupational therapy n = 4, dietetics n = 3, speech-language pathology n = 2) [46,50,51,53,54,59,60,63,64,67-69,71,75,76]. Seventeen studies examined multiple KT interventions (pharmacy n = 7, physiotherapy n = 9, occupational therapy n = 2) [45,47-49,52,55-58,61,62,65,66,70,72-74]. Following the *EPOC* classification scheme, the predominant single KT intervention was educational meetings (n = 11) [46,51,53,54,59,63,64,69,71,75,76], followed by educational materials (n = 2) [50,67], educational outreach visits (n = 1) [68], and a financial intervention (n = 1) [60]. The studies employing multiple interventions all contained at least one education-related component. Nine of these studies used education interventions exclusively: educational meeting and educational material (n = 7) [47,48,56,61,62,65,73]; educational outreach visit and educational material (n = 1) [52]; educational meeting, educational outreach visit, and educational material (n = 1) [72]. The remaining eight studies employing multiple interventions represented the following combinations: educational meeting and reminders (n = 2) [57,70]; educational material and mass media (n = 1) [45]; educational meeting and local opinion leaders (n = 1) [74]; educational meeting, educational material, and reminders (n = 1) [66]; educational meeting, educational outreach visit, and audit and feedback (n = 1) [49]; educational materials, educational outreach visit, and mass media (n = 1) [55]; educational meeting, educational material, and local opinion leaders (n = 1) [58]. Table 3 describes the KT interventions of the studies in greater detail.

### KT interventions by profession

When the KT interventions were examined by profession, educational meetings were used most often in dietetics (n = 3; 100% of dietetics studies) [46,53,63]; occupational therapy (n = 3; 50% of occupational therapy studies) [59,75,76]; pharmacy (n = 3; 25% of pharmacy studies) [51,54,69]. Pharmacy studies employed the widest range of interventions, including multiple interventions (n = 7; 58%) [45,49,55-58,62,65], educational meetings (n = 2; 16%) [54,69], educational material (n = 1; 8%) [50], and financial intervention (n = 1; 8%) [60]. Two speech-language pathology studies were included in this review; the KT interventions were educational meetings [71] and educational outreach visits [68].

### Outcomes

#### Outcome categories

The studies assessed outcomes at different levels; therefore, we applied the *EPOC* classification scheme (*i.e.*, professional/process outcomes, patient outcomes, and economic outcomes). Of the 32 included studies, the primary outcomes were professional/process outcomes (n = 25) [46,47,49-59,63-68,70,71,73-76], patient outcomes (n = 4) [48,60,62,72], and economic outcomes (n = 2) [61,69]. One study identified both professional/process and patient outcomes as primary outcomes (*e.g.*, professional/patient communication and patient education) [45].

### Outcomes by profession

The dietetics, occupational therapy, and speech-language pathology studies used only professional/process outcomes to assess KT interventions. The pharmacy and physiotherapy studies identified a wider range of outcomes. The outcomes of the pharmacy studies were: professional/process (n = 8) [49-51,54-57,65], patient (n = 2) [60,62], economic (n = 1) [69], and combination professional/process and patient outcomes (n = 1) [45]. The outcomes of the physiotherapy studies were: professional/process (n = 8) *e.g.*, 48,52,58,64,70,73-76], patient (n = 2) [47,72], and economic (n = 1) [61].

### Outcomes by KT intervention

The studies using educational meetings as the single KT intervention used professional/process outcomes (n = 10) [46,51,53,54,59,63,64,71,75,76] and economic outcomes (n = 1) [69].

### Interventions

#### Intervention effects in quantitative research studies: primary outcomes

Some studies did not clearly identify a primary outcome from a host of outcomes measured. Further, it was typical for an identified primary outcome to be measured in multiple ways. At times, this practice led to mixed results within the main outcome(s). To address this, we looked for consistency (*e.g.*, all positive or all negative effects) within the results. We categorized studies that reported both positive and negative effects for the same outcome as having ‘mixed effect.’ Studies that had all positive or all negative effects for the same outcome were categorized as ‘consistent effect.’ Studies in which the results were not clearly linked to the identified outcome(s) were classified as ‘unclear,’ and studies in which there were no comparative statistics provided or results were not reported for the identified outcome(s) were classified as ‘not done’.

As described in Table 2, less than a third of the quantitative studies showed a consistent effect on primary outcome measures (n = 8) [47,48,50,52,64,65,71,72]. Five studies could not be classified as consistent or mixed effect on primary outcome measures: unclear (n = 2) *i.e.*,58,70], not done (n = 3) [57,59,67].

**Table 2:** 

**First author (Year) Country**	**Study design (Sample size)**	**Single/ multiple intervention**	**EPOC intervention(s)**	**Type of targeted behaviour (EPOC) (specific behaviour targeted)**	**Main outcomes (EPOC)**	**Effect on main outcomes Consistent (C), Mixed (M), Unclear, Not done**
**Pharmacy studies**						
Hoffmann, W, et al. [62] (2008) Germany	Randomized controlled trial (112 pharmacies)	Multiple	Professional- educational meetings	Procedures (intensified structured counselling)	Patient	M
			Professional- educational material			
Hirsch, JD, et al. [60](2009) USA	Retrospective cohort study (10 pharmacies)	Single	Financial: provider-fee for service	General management of a problem (medication therapy management services)	Patient	M^1^
Munroe, WP, et al. [69](1997) USA	Retrospective cohort study (8 pharmacies)	Single	Professional- educational meetings	General management of a problem (patient-focused pharmacist intervention)	Economic	M
Bracchi, RCG, et al. [50](2005) UK	Non-concurrent cohort study (261 Pharmacists)	Single	Professional- educational material	Procedures (adverse drug reaction reporting)	Professional/process	C (significant positive effect)
Dualde, E, et al. [54](2009) Spain	Non-concurrent cohort study (190 Pharmacists)	Single	Professional- educational meetings	Procedures (pharmacotherapy follow-up services)	Professional/process	M
Airaksinen, M, et al. [45] (1998) Finland	Before-after study (7 pharmacies)	Multiple	Professional- educational materials	Procedures (patient counselling)	Professional/process	M
			Professional- mass media		Patient	
Benrimoj, SI, et al. [49](2007) Australia	Before-after study (40 pharmacies)	Multiple	Professional- educational meetings	Procedures (standards of practice for handling non-prescription meds)	Professional/process	M
			Professional- educational outreach visits			
			Professional- audit and feedback			
Egen, V, et al. [55](2003) Germany	Before-after study (42 Pharmacists)	Multiple	Professional- mass media Professional- educational outreach visits Professional- educational materials	Patient education (promoting prophylaxis)	Professional/process	M^2^
Fjortoft, N, et al. [56](2003) USA	Before-after study (49 Pharmacists)	Multiple	Professional- educational meetings	General management of a problem (lipid management and hypertension services)	Professional/process	M
			Professional- educational material			
Fjortoft, N, et al. [57](2007) USA	Before-after study (33 Pharmacists)	Multiple	Professional- educational meetings	Other (preceptor leadership)	Professional/process	Not done^3^
			Professional- reminders			
Martin, BA, et al. [65](2010) USA	Before-after study (25 Pharmacists)	Multiple	Professional- educational meetings	General management of a problem (tobacco cessation)	Professional/process	C^4^ (significant positive effect)
			Professional- educational material			
Brooks, V. G. [51] (2001) USA	Cross-sectional study (213 Pharmacists)	Single	Professional- educational meetings	Other (broad education activities)	Professional/process	M
**Physiotherapy Studies**						
Bekkering, GE, van Tulder MW, et al. [48] (2005) The Netherlands	Randomized controlled trial (113 Physiotherapists)	Multiple	Professional- educational materials	General management of a problem (clinical guidelines for low back pain)	Patient	C (non-significant)
			Professional- educational meetings			
Bekkering, GE, Hendricks, HJM, et al. [47] (2005) The Netherlands	Randomized controlled trial (113 Physiotherapists)	Multiple	Professional- educational materials	General management of a problem (clinical guidelines for low back pain)	Professional/process	C (significant positive effect)
			Professional- educational meetings			
Hoeijenbos, M, et al. [61] (2005) The Netherlands	Randomized controlled trial (113 Physiotherapists)	Multiple	Professional- educational materials (2)	General management of a problem (clinical guidelines for low back pain)	Economic	M^5^
			Professional- educational meetings			
Rebbeck, T, et al. [72] (2006) Australia	Randomized controlled trial (27 Physiotherapists)	Multiple	Professional- educational meetings	General management of a problem (clinical guidelines for acute whiplash)	Patient	C^6^ (non-significant)
			Professional- educational outreach visits			
			Professional- educational materials (3)			
Stevenson, K, et al. [74] (2006) UK	Randomized controlled trial (30 Physiotherapists)	Multiple	Professional- educational meetings	General management of a problem (clinical management of patients with low back pain)	Professional/process	M
			Professional- local opinion leaders			
Kerssens, JJ, et al. [64] (1999) The Netherlands	Interrupted time series (without comparison group) (19 Physiotherapists)	Single	Professional- educational meetings	Patient education	Professional/process	C^7^ (non-significant)
Brown, CJ et al. [52] (2005) USA	Cross-sectional study (94 Physiotherapists)	Multiple^8^	Professional- educational outreach visits	Illness prevention (fall prevention)	Professional/process	C^9^ (significant positive effect)
			Professional- educational material (2)			
Gross, DP, et al. [58] (2009) Canada	Cross-sectional study (241 Physiotherapists)	Multiple	Professional- educational meetings	General management of a problem (work disability prevention)	Professional/process	Unclear^10^
			Professional- educational materials			
			Professional- local opinion leaders			
Schreiber, J, et al. [73] (2009) Canada	Qualitative - participatory action research (5 Physiotherapists)	multiple	Professional- educational meetings	Other (evidence-based practice)	Professional/process	Sustained positive attitude and beliefs about evidence-based practice.
			Professional- educational material (2)			
						Variable performance related to evidence-based practice knowledge and behaviours.
**Physiotherapy and Occupational Therapy Studies**						
Nikopoulou-Smyrni, P, et al. [70] (2007) UK	Randomized controlled trial (4 Physiotherapists and 4 Occupational Therapists)	Multiple	Professional- educational meetings	Procedures (application of new clinical reasoning model)	Professional/process	Unclear
			Professional-reminders			
Tripicchio, B, et al. [75] (2009) USA	Before-after study (24 Therapists)	Single	Professional- educational meetings	Professional-patient communication (OPN Method)	Professional/process	M
**Occupational Therapy Studies**						
McCluskey, A, et al. [66] (2005) Australia	Before-after study (114 Occupational Therapists)	Multiple	Professional- educational meetings	Other (evidence-based practice)	Professional/process	M
			Professional- educational material			
			Professional- reminders^11^			
Hammond, A, et al. [59] 2005) UK	Cross-sectional study (48 Occupational Therapists)	Single	Professional- educational meetings	General management of a problem (joint protection)	Professional/process	Not done^12^
McKenna, K, et al. [67] (2005) Australia	Cross-sectional study (213 Occupational Therapists)	Single	Professional- educational material	Other (evidence-based practice)	Professional/process	Not done
Vachon, B, et al. [76] (2009) Canada	Qualitative - grounded theory (8 Occupational Therapists)	Single	Professional- educational meetings	Other (evidence-based decision making)	Professional/process	Participants developed their ability to use 6 different types of reflective thinking, which brought about perspective changes in their clinical decision-making process and sometimes lead to application in professional practice. Perspective changes were not achieved at the same pace/level by all participants.
**Dietetics Studies**						
Banz, M, et al. [46] (2004) USA	Randomized controlled trial (172 Dieticians)	Single	Professional- educational meetings	Patient education	Professional/process	M
Brug, J, et al. [53] (2007) The Netherlands	Randomized controlled trial (37 Dieticians)	Single	Professional- educational meetings^13^	Procedures (counselling style)	Professional/process	M^14^
Johnson, ST, et al. [63] (2007) Canada	Cross-sectional study (103 Dieticians)	Single	Professional- educational meetings	Patient education	Professional/process	M
**Speech-Language Pathology Studies**						
Pennington, L, et al. [71] (2005) UK	Randomized controlled trial (34 Speech-Language Pathologists)	Single	Professional- educational meetings	Other (evidence-based practice)	Professional/process	C^15^ (non-significant)
Molfenter, SM, et al. [68] (2009) Canada	Qualitative - not clear (4 Speech-Language Pathologists)	Single	Professional- educational outreach visits^16^	General management of a problem (Dysphagia)	Professional/process	The intervention enhanced the participants learning and allowed them to offer a greater quantity and variety of services to their patients.

^1^Secondary outcomes measured at the level of economics with mixed effect.

^2^Secondary outcomes measured at the level of the patient with mixed effect.

^3^Secondary outcomes identified at the level of the professional/process with positive effect; however, comparative statistics were not done.

^4^Secondary outcomes measured at the level of the professional/process with a consistent, statistically significant, positive effect and Tertiary outcomes measured at the level of the professional/process with consistent effect (non-significant).

^5^Secondary outcomes measured at the level of the patient with mixed effect.

^6^Secondary outcomes measured at the level of the professional/process with mixed effect and the level of economics with consistent effect (non-significant).

^7^Secondary outcomes measured at the level of the patient with consistent effect (non-significant).

^8^Participants exposed to other interventions (not described or measured in this research report) as part of an ongoing, community-wide effort to translate research evidence into practice.

^9^Secondary outcomes were identified at the level of the professional/process; however, results were not reported.

^10^Secondary outcomes measured at the level of economics with consistent effect (non-significant).

^11^The intervention also included an optional educational outreach visit; however, data was not collected or reported on this aspect of the intervention.

^12^Secondary outcome measured at the level of the professional/process with mixed effect.

^13^The intervention had an additional component termed ‘on demand feedback and advice’ that cannot be classified within the EPOC frameworkiple (n=1 (n=21.

^14^Secondary outcomes measured at the level of the patient with mixed effect.

^15^Secondary outcomes measured at the level of economics with consistent effect (non-significant).

^16^The intervention had an additional component termed ‘monitor progress, provide assistance on as needed basis via email, telephone, in-person’ that cannot be classified within the EPOC framework.

### Studies with mixed effects

The majority of the quantitative studies (n = 16) demonstrated ‘mixed effects’ on primary outcome measures [45,46,49,51,53-56,60-63,66,69,74,75]. The research designs of the studies demonstrating mixed effects were: randomized controlled trial (n = 5) [46,53,61,62,74], retrospective cohort study (n = 2) [60,69], non-concurrent cohort study (n = 1) [54], before-after study (n = 6) [45,49,55,56,66,75], and cross-sectional study (n = 2) [51,63].

### Studies with consistent effects

Eight studies demonstrated a consistent effects on primary outcomes; however, four studies demonstrated effects that were not statistically significant [48,64,71,72]. The remaining four studies demonstrated a statistically significant, positive effects on primary outcomes [47,50,52,65]. Bekkering *et al*. [47] conducted a randomized controlled trial that examined a group of physiotherapists (n = 113) attempting to implement clinical guidelines for low back pain by using multiple, education-only interventions (*i.e.*, educational material and educational meeting). The physiotherapists in this study reported a statistically significant increase in adherence to the main recommendations of the guidelines. Bracchi *et al*. [50] conducted a non-concurrent cohort study that examined a group of pharmacists (n = 261) attempting to change adverse drug reaction reporting procedures by using educational material as a single KT intervention. In this study, the results from the control year were compared to the results from the study year and the study region was compared to a control region during both years. As a result, a statistically significant increase in the number of adverse drug reaction reports and the number of ‘appropriate’ adverse drug reaction reports were reported in the study region. Brown *et al*. [52] conducted a cross-sectional study that examined a sample of physiotherapists (n = 94) attempting to change fall prevention strategies by using multiple, education-only interventions (*i.e.*, educational outreach visits and educational material). The physiotherapists participating in this study reported a statistically significant increase in the frequency of self-reported fall prevention practice behaviours compared to one year prior to the study intervention. Martin *et al*. [65] conducted a before-after study that examined a group of pharmacists (n = 25) attempting to change tobacco cessation counseling by using multiple, education-only interventions (*i.e.*, educational meetings and educational material). The pharmacists in this study reported a statistically significant increase in self-efficacy measures and current skill measures for the 5A’s counseling process post-intervention.

### Intervention effects in quantitative research studies: secondary outcomes

Secondary outcomes were measured and reported in 12 of 29 quantitative studies [52,53,55,57-61,64,71,72]. The secondary outcomes were: professional/process outcomes (n = 4) [52,57,59,72], patient outcomes (n = 4) [53,55,61,64], economic outcomes (n = 3) [58,60,71]. One study measured both professional/process and economic secondary outcomes [72]. Of these studies, one study demonstrated a consistent, statistically significant, positive effect [65], and four demonstrated consistent, statistically non-significant effects on secondary outcome measures [58,64,71,72]. Six studies showed ‘mixed effects’ [53,55,59-61,72]. Two studies did not provide comparative statistics and were classified as ‘not done’ [52,57].

### Intervention effects by profession

When the intervention effects were examined by profession, two disciplines contained the four quantitative studies that consistently demonstrated consistent, statistically significant, positive effects on primary outcome measures: pharmacy (n = 2) [50,65] and physiotherapy (n = 2) [47,52]. The studies with consistent, non-significant effects on primary outcome measures were as follows: physiotherapy (n = 3) [48,64,72] and speech-language pathology (n = 1) [71]. All of the dietetics studies (n = 3) demonstrated mixed effects [46,53,63]. Mixed effects were also reported for the primary outcome measures in the following professions: pharmacy (n = 9) [45,49,51,54-56,60,62,69]; physiotherapy (n = 3) [61,74,75]; occupational therapy (n = 2) [66,75].

### Intervention effects of randomized controlled trials

Ten RCTs were included in this review representing the following professions: physiotherapy (n = 6) [47,48,61,70,72,74]; dietetics (n = 2) [46,53]; pharmacy (n = 1) [62]; speech-language pathology (n = 1) [71]. These studies employed a variety of KT interventions: multiple, education-only (n = 5) [47,48,61,62,74]; single educational meeting (n = 3) [46,53,71]; multiple interventions (*i.e.*, education intervention and another non-education intervention) (n = 2) [70,72]. Five studies demonstrated mixed effects on primary outcomes [46,53,61,62,74], four studies demonstrated consistent effects on primary outcomes [47,48,71,72], and the effects of the intervention on the primary outcome in the remaining study was unclear [70]. Of the four studies demonstrating consistent effects on the primary outcomes, three studies demonstrated non-significant effects [47,71,72] and the remaining study demonstrated statistically significant, positive effects on the primary outcome [48].

### Intervention evaluation in qualitative research studies

The three qualitative studies included in this review represented the following professions: speech-language pathology, physiotherapy, and occupational therapy. Molfentner *et al*. [68] conducted a qualitative study using the knowledge-to-action (KTA) process model framework to address an identified KTA gap in dysphagia rehabilitation practices for speech-language pathologists (n = 4). This study employed a single KT intervention (*e.g.* educational outreach visits), and after conducting post-intervention interviews with the study participants, it was determined that ‘the intervention not only enhanced their learning, but also allowed them to offer a greater quantity and variety of services to their patients. Clinicians reported that having hands-on training by a research S-LP was more effective than a lecture on the same topic.’ Schreiber *et al*. [73] conducted a qualitative, participatory action research study to identify, implement, and evaluate the effectiveness of strategies to incorporate research evidence into clinical decision making in physiotherapy (n = 5). Gathering data through semi-structured interviews, this study reported that multiple, education-only interventions (*e.g.* educational meetings and educational materials) gave rise to themes that included ‘sustained positive attitudes and beliefs about evidence-based practice, variable implementation of the strategies developed during the initial collaboration phase, variable performance for individual goals; persistent barriers, including a lack of time and a lack of incentives for evidnce-based practice activities; and a desire for user-friendly evidence-based clinical practice guidelines.’ Vachon *et al*. [76] conducted a qualitative study using grounded theory to describe how rehabilitation professionals use reflective learning to incorporate research evidence into clinical decision making and to identify factors that influenced the reflective learning process. This study employed a single KT intervention (*e.g.* educational meetings) with a population of occupational therapists (n = 8). Data were collected via meeting videotapes, transcripts, written critical incident descriptions, reflective journals, and the facilitator’s notes and summaries. Through this intervention, ‘the participants developed their ability to use different types of reflective thinking, which brought about perspective changes… however, perspective changes were not achieved at the same pace or the same level by all participants. Some personal and contextual factors were found to influence the participants’ ability to learn reflectively’.

These three studies employed both single and multiple education-related KT interventions targeting an allied health professional’s general management of a problem [68] or evidence-based practices [73,76]. The behaviour changes in all three studies were evaluated using the professional/process outcomes. While there were some encouraging findings, such as sustained positive attitudes and beliefs [68], ability to use different types of reflective thinking [76], and enhanced learning and services [73], all of the studies acknowledged variable practice changes related to the targeted behaviours.

### Published intervention reporting

The quality and detail of the reporting of the KT interventions varied widely between the 32 study reports; therefore, the published intervention descriptions were compared to the *WIDER Recommendations to Improve Reporting of the Content of Behaviour Change Interventions*[39], which were developed in 2009. While a small number of studies met three of the four criteria, none of the 32 studies satisfied all four of the *WIDER Recommendations*. However, it is important to note that some authors reported more intervention details than others (Table 3). Many of the studies described components of the first recommendation, such as descriptions of intervention recipients, the intervention setting, the mode of delivery, and the intensity and duration of the intervention. Nevertheless, most did not provide a full and detailed description, which would include a description of the characteristics of the individuals delivering the intervention, the adherence/fidelity to delivery protocols, or a detailed description of intervention content. A number of studies provided an outline of the intervention objectives. In relation to the second recommendation, four studies described in detail the clarification of assumed change process and design principles [49,64,68,76]. Several studies included a description of a theoretical framework informing their research, the rationale behind and impetus for the intervention, and the behaviour that the intervention was intended to change; however, most did not describe the development of the intervention, the change techniques used in the intervention, or the causal processes targeted by these change techniques. Only five studies fulfilled the third recommendation of providing access to intervention manuals or protocols within the article or in separate publications [49,58,62,64,67]. Most studies were exempt from the fourth recommendation because the study designs did not include a control group (n = 17) [45,49,52,54-59,65-68,71,73,75,76] or active control conditions (n = 12) [46-48,50,51,53,60-64]. None of the three studies with active controls satisfied this criteria [70,72,74]. Table 3 contains an overview of the *WIDER Recommendations*[39] in relation to each of the included studies.

**Table 3:** 

**First author** (**Year**)	**WIDER recommendations to improve reporting of the content of behaviour change interventions**			
	**Detailed description of intervention**	**Clarification of assumed change process and design principles**	**Access to intervention Manuals**/ **Protocols**	**Detailed description of active control conditions**
	(**Y**/**N**)	(**Y**/**N**)	(**Y**/**N**)	(**Y**/**N**)
**Pharmacy Studies**				
Hoffmann, W, *et al*. (2008)	N	N	Y	No active control
Hirsch, JD, *et al*. (2009)	N	N	N	No active control
Munroe, WP, *et al*. (1997)	N	N	N	No active control
Bracchi, RCG, *et al*. (2005)	N	N	N	No active control
Dualde, E, *et al*.(2009)	N	N	N	No control group
Airaksinen, M, *et al*. (1998)	N	N	N	No control group
Benrimoj, SI, *et al*. (2007)	N	Y	Y	No control group
Egen, V, *et al*.(2003)	N	N	N	No control group
Fjortoft, N, *et al*. (2003)	N	N	N	No control group
Fjortoft, N, *et al*. (2007)	N	N	N	No control group
Martin, BA, *et al*. (2010)	N	N	N	No control group
Brooks, VG, *et al*. (2001)	N	N	N	No active control
**Physiotherapy Studies**				
Bekkering, GE, van Tulder MW, *et al*. (2005)	N	N	N	No active control
Bekkering, GE, Hendricks, HJM, *et al*. (2005)	N	N	N	No active control
Hoeijenbos, M, *et al*. (2005)	N	N	N	No active control
Rebbeck, T, *et al*. (2006)	N	N	N	N
Stevenson, K, *et al*. (2006)	N	N	N	N
Kerssens, JJ, *et al*. (1999)	N	Y	Y	No active control
Brown, CJ *et al*. (2005)	N	N	N	No control group
Gross, DP, *et al*. (2009)	N	N	Y	No control group
Schreiber, J, *et al*. (2009)	N	N	N	No control group
**Physiotherapy & Occupational Therapy Studies**				
Nikopoulou-Smyrni, P, *et al*. (2007)	N	N	N	N
Tripicchio, B, *et al*. (2009)	N	N	N	No control group
**Occupational Therapy Studies**				
McCluskey, A, *et al*. (2005)	N	N	N	No control group
Hammond, A, *et al*. (2005)	N	N	N	No control group
McKenna, K, *et al*. (2005)	N	N	Y	No control group
Vachon, B, *et al*. (2009)	N	Y	N	No control group
**Dietetics Studies**				
Banz, M, *et al*. (2004)	N	N	N	No active control
Brug, J, *et al*. (2007)	N	N	N	No active control
Johnson, ST, *et al*. (2007)	N	N	N	No active control
**Speech**-**Language Pathology Studies**				
Pennington, L, *et al*. (2005)	N	N	N	No control group
Molfenter, SM, *et al*. (2009)	N	Y	N	No control group

### Methodological quality

We assessed the 29 quantitative studies and three qualitative studies using separate tools (Additional File 7) [41], six quantitative studies received a moderate rating [48,54,55,62,74,75], and 23 studies received a weak rating [45-47,49-53,56-61,63-67,69-72]. None of the 29 quantitative studies received a strong rating. Additionally, of the four studies that demonstrated consistent, significant positive effects on the primary outcomes illustrating that the KT interventions had effectively changed the identified behaviours, it is important to note that all received a weak rating using this methodological quality assessment tool.

Based on the *Quality Assessment Tool for Qualitative Studies*[43], with higher values denoting higher study quality, one qualitative study was given a rating of five [76] and the other two studies were rated two [73] and one [68], respectively.

### Summary of changes from the study protocol

The following items were changed during the research process; therefore, the study protocol [33] should be adjusted to reflect these changes: the inclusion criteria was clarified according to the *EPOC Data Collection Checklist*[37] (Addition File 2); the data extraction process was modified to include a research design algorithm [38] (Additional File 3) to be used in place of the study design component of the *EPOC Data Collection Checklist*; and the methodological quality assessment tool for qualitative studies that was described in the protocol was replaced with the *Quality Assessment Tool for Qualitative Studies*[43] (Additional File 6).

## Discussion

This systematic review identified 32 studies that investigated a variety of KT strategies to put research into practice in the allied health disciplines. This review complements the extant research on broad approaches to put research into practice in particular disciplines, such as nursing *e.g.*, [10], [11] and the reviews of specific KT strategies (*e.g.*, audit and feedback, financial incentives) with a focus on medicine *e.g.*, [7-9]. Until now, reviews completed by Hakkennes and Dodd [19] and Menon *et al*. [20] provided the most comprehensive data on KT strategies in allied health; however, our review built on this existing research in several important ways. First, our review explored all types of interventions or approaches (*i.e.*, a variety of professional and financial interventions) to put research into professional practice. Second, our review had a concise conceptualization of allied health that reflected typical, acute care health environments in Canada (*i.e.*, containing five professions). Third, our review was inclusive of all research designs, which led to the inclusion of 32 studies across the five professions and reflects the largest review in this area conducted to date.

Our findings make several important contributions to KT science, specifically in terms of the allied health professions, in three important ways: identifying a considerable reliance on educational interventions to change practice behaviour; clarifying the impact of outcome reporting bias; and innovatively employing the *WIDER Recommendations*[39] as a framework to identify components missing from current research reporting behaviour change interventions. These three contributions will frame the following discussion.

A number of studies [4-6,26,78-80] have clearly demonstrated that education alone has a limited impact on changing healthcare professionals’ clinical practices and behaviours. In this review, education-only approaches were frequently employed (n = 23) and our findings suggest that educational approaches on their own did not propel the desired provider practice change. Interestingly, 15 studies in this review employed a single KT intervention, with 11 of these studies using educational meetings. Results from the studies were inconsistent, with 16 of 29 studies demonstrating ‘mixed effects’ on the primary outcome evaluating the KT interventions. Eight of the quantitative studies demonstrated ‘consistent effects’ with the reported variables; however, four of these studies demonstrated consistently non-significant effects. All of the studies that illustrated consistently non-significant effects employed education-only interventions, with one-half employing single, educational meetings. These findings suggest a potential area warranting further exploration, that is, the exclusive provision of knowledge through educational interventions may not be adequate to change behaviour in the allied health professions. These findings stress the need for KT researchers to consider how other types of KT interventions may be used foster change. However, due to the poor reporting of the KT interventions, it is difficult to determine more specifically what intervention aspects contribute to behaviour change or lack thereof. It is important to note that the high frequency of educational interventions is not exclusive to the allied health professions, and a similar trend is seen in nursing [11] and in the guideline implementation literature, which is largely dominated by physicians [6].

Categorically speaking, educational interventions consist of a broad range of activities (*e.g.*, educational materials, large-scale meetings, small scale meetings, outreach visits, *et al*.) intended to increase knowledge and skills with the expectation that new information will facilitate behaviour change [81]. In many cases, the decision to change provider behaviour may not be straightforward, because it involves more than convincing healthcare professionals of the strength and rigor of the research informing the innovation. Often, provider behaviour change requires persuasion at multiple levels (*e.g.*, healthcare professional, department decision-makers, *et al*.) and the allocation of significant resources to support the change. Individual healthcare professionals cannot simply decide to change their clinical practice and decision making to be aligned with an innovation (*e.g.*, research-based clinical pathway, research-based protocol). Allied health professionals work within complex organizational structures and frequently as members of interprofessional teams; thus, behaviour change is complex due to a number of competing factors (many of which may be beyond their immediate control). As well, these competing factors that are beyond the typical ‘scope’ of an allied healthcare professionals’ practice suggest the need for KT researchers to consider the new possibilities, for example, institutional, organizational, or legislative. Our findings echo previous research that suggests that the effects of education on behaviour may be limited [5,78,80,82-85], but may represent a necessary ingredient or first step in the process of change.

In the studies reviewed, discrepancies between the outcomes described in the methods and results sections were common, and it was not always possible to reliably differentiate the primary outcome from secondary outcomes. This opacity was further compounded with some outcomes being measured using multiple tools and approaches. The resulting ambiguity led to substantial interpretation challenges. In the literature, the challenge of vague and incomplete reporting of outcomes has been referred to as outcome reporting bias, selective outcome reporting, and within-study selective reporting [82-86]. Generally speaking, outcome reporting bias refers to the selective reporting of some results but not others in publications [83]. There is emerging evidence that this is a common problem in the literature, yet it is a difficult problem to pinpoint [84]. Recently, studies have examined the extent and nature of outcome reporting bias through a review of publications and follow up surveys with authors [83] and comparison of protocols with published study reports [82] that, in some cases, were augmented with interviews with trialists [85]. These studies have identified that outcome reporting bias is high in the literature [82,86]. The implications of outcome reporting bias for healthcare research are significant. For example, an intervention may be considered to be of more value than it merits; on the other hand, not reporting all outcomes could lead to the use of ineffective or potentially harmful interventions. Other implications include a tendency to overestimate the effects of interventions because the primary outcome is the basis for sample size determination. Thus, when the primary outcome is replaced by a secondary outcome, erroneous results may result due to inadequate sample size [85]. Our findings echo the need to limit outcome reporting bias in publications and to increase transparency in research reporting.

Interventions to change healthcare professionals’ behaviours are deemed to be effective if they make a difference in terms of the identified outcomes. Clear descriptions of the intervention procedures are integral to understanding why an intervention works and facilitate replication of successful interventions. There is a burgeoning discussion of the extent of poor intervention reporting [26], with reports suggesting that in a review of 1,000 behaviour change outcome studies only 5% to 30% of the experimental studies described the intervention in adequate detail [87-90]. Clearly delineating intervention components, relationships between components, and the outcomes are essential to future development and implementation of the intervention [26] and lead to future contributions to science and practice in terms of more confidence in large-scale replication. In response to poor intervention reporting, the *WIDER Recommendations*[39] were developed to provide guidance. We applied the *WIDER Recommendations* to the studies in our review and our findings mirrored previous earlier documented reports [87-90]. All studies failed to meet the four critical criteria; as a result, we highlight the need to publish study protocols, make intervention descriptions and protocols publically available, and report interventions using the *WIDER Recommendations*.

The strengths of this systematic review also represent its weaknesses. Methodological inclusivity was a key element of this study, which allowed us to review a large number of diverse studies; however, due to the heterogeneity of the 32 studies we were not able to conduct meta-analysis in order to determine definitive practice recommendations. As we stressed earlier, another limitation of our findings is that much of the literature included in this review was of a moderate or weak quality.

## Conclusions

Our findings provide the first systematic overview of KT strategies used in allied health professionals’ clinical practice, as well as a foundation to inform future KT interventions in allied healthcare settings. The findings of this review reveal an over-reliance on educational strategies without a clear effect on the intended outcomes; therefore, it is recommended that researchers establish a clear connection between the intended behaviour change and the KT interventions used to foster this change. Additionally, due to the nature and scope of work within distinct professions, it is important to note that the success of KT interventions does not necessarily transfer from one profession to another.

For those charged with the task of putting research into practice, it is important to note that while educational interventions are the most common KT strategies in allied health, there is great variation in approaches to these interventions. Additionally, these KT interventions are not reported in enough detail to be replicable and the effects of these interventions are equivocal.

For researchers and professionals tasked with ensuring that healthcare practices reflect the best available research evidence, it is important to be aware of the variability of individual interventions within the broader *EPOC* classification scheme of intervention categories [37]. This review demonstrates that the most common KT strategy used in allied health is educational meetings; however, this category reflects a group learning situation that encompasses both interactive and didactic educational strategies. More research is required to examine the nuances of this *EPOC* classification scheme intervention category.

## Abbreviations

KT: Knowledge Translation; EPOC: Cochrane Effective Practice and Organisation of Care Review Group; WIDER: Workgroup for Intervention Development and Evaluation Research.

## Competing interests

The authors declare that they have no competing interests.

## Authors’ contributions

SDS conceptualized this study and secured study funding from the Canadian Institutes for Health Research (CIHR). She designed and led this study. LA and KO coordinated the study team and the study itself; both assisted with the study design. The remaining authors assisted with the study design. In addition, DMD and LH provided methodological consultation. As well, KKB was the principal knowledge user for this study. All authors contributed to manuscript drafts and reviewed the final manuscript.

## Supplementary Material

Additional file 1 A Systematic Review of Knowledge Translation Strategies used in Allied Health Professions. Click here for file

Additional file 2 Cochrane Effective Practice and Organisation of Care Review Group. Click here for file

Additional file 3 Testing a tool for the classification of study designs in systematic reviews of interventions and exposures showed moderate reliability and low accuracy. Click here for file

Additional file 4 WIDER Recommendations to Improve Reporting of the Content of Behaviour Change Interventions. Click here for file

Additional file 5 Component ratings. Click here for file

Additional file 6 Quality assessment tool for qualitative studies. Click here for file

Additional file 7 Assessment of 29 quantitative studies and three qualitative studies using separate tools. Click here for file
