# Supplementary material for: Systematic review of knowledge translation strategies in the allied health professions
Source: Implement Sci. 2012 Jul 25;7:70. doi: 10.1186/1748-5908-7-70 (PMC3780719; doi:10.1186/1748-5908-7-70)
Supplement: Additional file 1 — A Systematic Review of Knowledge Translation Strategies used in Allied Health Professions. [file 1748-5908-7-70-S1.doc]

**Title: A Systematic Review of Knowledge Translation Strategies used in Allied Health Professions**

**Database:** MEDLINE

**Search name:** KT – allied professionals-1| MEDLINE 16Mar10

**Notes:** limits: English only, 1985-present

**Date searched:** 16Mar10

**Results:**  1004

| 1. exp Occupational Therapy/  2. (occupational adj therapist*).tw.  3. ((physical or occupational) adj2 therapy adj assistant).tw.  4. exp "Physical Therapy (Specialty)"/  5. (physical adj therapist*).tw.  6. physiotherapist*.tw.  7. exp Speech-Language Pathology/  8. (speech adj2 (therapist* or pathologist*)).tw.  9. exp dietetics/  10. (dietitian* or dietician*).tw.  11. (diet* adj2 techn*).tw.  12. exp Pharmacy/  13. Pharmacy Service, Hospital/  14. exp Pharmacists/  15. exp Pharmacists' Aides/  16. pharmacist*.tw.  17. (pharmacy adj (technologist* or technician*)).tw.  18. or/1-17  19. exp Audiovisual Aids/  20. exp Pamphlets/  21. education/  22. exp Teaching Materials/  23. exp Clinical Protocols/  24. exp practice guidelines as topic/  25. exp critical pathways/  26. exp inservice training/  27. exp consultants/  28. exp Staff Development/  29. exp Health Knowledge, Attitudes, Practice/  30. exp Reminder Systems/  31. exp Clinical Competence/  59. ((predisposing or enabling or reinforcing) adj factor*).tw.  60. ((support or impede) adj change*).tw.  61. (behavio?r adj2 change*).tw.  62. (knowledge adj2 (utilization or utilisation or uptake or transfer* or implementation or dissemination or diffusion* or translation)).tw. | 32. exp Education, Continuing/  33. professional development/  34. exp Professional Role/  35. exp Professional Competence/  36. exp Guideline Adherence/  37. (adherence adj3 guidelines).tw.  38. exp "Attitude of Health Personnel"/  39. exp Evidence-Based Practice/  40. exp evidence-based medicine/  41. (workshop* or seminar* or training or in-service).tw.  42. exp "Process Assessment (Health Care)"/  43. exp "Outcome and Process Assessment (Health Care)"/  44. exp "Delivery of Health Care"/  45. exp program development/mt  46. exp Health Plan Implementation/mt, og [Methods, Organization & Administration]  47. (journal adj club).ti,ab.  48. exp Quality Assurance, Health Care/  49. organizational case studies/  50. (champion* adj change*).tw.  51. (change adj2 agent*).tw.  52. ((facilitat* or coordinat*) adj2 change*).tw.  53. or/19-52  54. exp motivation/  55. exp Self Efficacy/  56. exp Organizational Innovation/  57. exp "diffusion of innovation"/  58. ((research or evidence or guideline*) adj3 (implementation or utilization or utilisation or diffusion or translation)).tw.  63. (increase adj2 implementation).tw.  64. (implementation adj2 (program or strategy or strategies)).tw.  65. decision making/  66. or/54-65  67. and/18,53,66  68. limit 67 to (english language and yr="1985 -Current") |
| --- | --- |

**NOTES:**

The highlighted yellow lines represent the three areas of our search in the strategy above:

Line 18 = allied health professions

Line 53 = various types and formats of educational components/activities

Line 66 = knowledge translation

Line 67 represents the search results by combining the above the above 3 search areas with AND. After limits were applied in line 61, we are left with 755 results

**Database:** CINAHL

**Search name:** KT – allied professionals-CINAHL

**Notes:** limits: English only, 1985-present

**Date searched:** 17Mar10

**Results:**  973

| **#** | **Query** |
| --- | --- |
| S64 | S37 and S55 and S16: english only, 1985-2010 |
| S63 | S37 and S55 and S16 |
|  |  |
| S55 | S38 or S39 or S40 or S41 or S42 or S43 or S44 or S45 or S46 or S47 or S48 or S49 or S50 or S51 or S52 or S53 or S54 |
| S54 | (MH "Decision Making+") or (MH "Decision Making, Clinical") or (MH "Decision Making, Organizational") |
| S53 | (MH "Program Implementation") or (MH "Systems Implementation") |
| S52 | TX implementation program* or TX implementation strategy or TX implementation strategies or TX Implementation plan |
| S51 | TX increase implementation |
| S50 | (MH "Behavioral Changes") or (MH "Transtheoretical Stages of Change Model") |
| S49 | TX behaviour change or TX behavior change |
| S48 | TX predisposing factor* or TX enabling factor* or TX reinforcing factor* |
| S47 | TX knowledge implementation or TX knowledge utilization or TX knowledge diffusion or TX knowledge translation or TX knowledge utilisation |
| S46 | TX guideline implementation or TX guideline utilization or TX guideline diffusion or TX guideline translation or TX guideline utilisation |
| S45 | TX evidence implementation or TX evidence utilization or TX evidence diffusion or TX evidence translation or TX evidence utilisation |
| S44 | TX research implementation or TX research utilization or TX research diffusion or TX research translation or TX research utilisation |
| S43 | (MH "Program Implementation") |
| S42 | (MH "Self-Efficacy") |
| S41 | (MH "Diffusion of Innovation") |
| S40 | (MH "Decision Making, Organizational") or (MH "Organizational Change") or (MH "Organizational Compliance") or (MH "Organizational Culture") |
| S39 | (MH "Attitude to Change") |
| S38 | (MH "Motivation") |
| S37 | S22 or S23 or S24 or S25 or S26 or S27 or S28 or S29 or S30 or S31 or S32 or S33 or S34 or S35 or S36 |
| S36 | TX champion* of change or TX change agent* or TX facilitator* of change or TX coordinator* of change |
| S35 | TI journal club or AB journal club |
| S34 | TX (workshop* OR seminar* OR training OR in-service) |
| S33 | (MH "Occupational Therapy Practice, Evidence-Based") or (MH "Physical Therapy Practice, Evidence-Based") or (MH "Professional Practice, Evidence-Based+") |
| S32 | TX adherence to guidelines |
| S31 | (MH "Guideline Adherence") |
| S30 | (MH "Professional Competence") |
| S29 | (MH "Professional Role") |
| S28 | (MH "Staff Development+") |
| S27 | (MH "Continuing Education Providers") or (MH "Education, Continuing+") |
| S26 | (MH "Clinical Competence+") |
| S25 | (MH "Reminder Systems") |
| S24 | (MH "Practice Guidelines") |
| S23 | (MH "Pamphlets") |
| S22 | (MH "Audiovisuals") |
| S16 | S1 or S2 or S3 or S4 or S5 or S6 or S7 or S8 or S9 or S10 or S11 or S12 or S13 or S14 or S15 |
| S15 | (MH "Pharmacy Technicians") |
| S14 | (MH "Pharmacists") |
| S13 | (MH "Pharmacy and Pharmacology") |
| S12 | (MH "Dietetic Technicians, Registered") |
| S11 | (MH "Dietitians") |
| S10 | (MH "Dietetics") |
| S9 | (MH "Speech-Language Pathologists") |
| S8 | (MH "Speech-Language Pathology Assistants") |
| S7 | (MH "Speech-Language Pathology") |
| S6 | (MH "Occupational Therapists") |
| S5 | (MH "Physical Therapists") |
| S4 | (MH "Occupational Therapy Assistants") |
| S3 | (MH "Physical Therapist Assistants") |
| S2 | (MH "Physical Therapy+") |
| S1 | (MH "Occupational Therapy+") |

**Title: A Systematic Review of Knowledge Translation Strategies used in Allied Health Professions**

**Database:** ERIC

**Search name:** KT – allied professionals-2 | ERIC 16Mar10

**Notes:** limits: english only, 1985-present

**Date searched:** 16Mar10

**Results:**  80

| 1. exp Occupational Therapy/  2. (occupational adj therapist*).tw.  3. ((physical or occupational) adj2 therapy assistant).tw.  4. exp Physical Therapy/  5. exp Speech Language Pathology/  6. exp Dietetics/  7. (dietitian or dietician).tw.  8. exp Pharmacy/  9. pharmacist*.tw.  10. (pharmacy adj (technologist* or technician*)).tw.  11. (physical adj therapist*).tw.  12. physiotherapist*.tw.  13. or/1-12  14. exp Audiovisual Aids/  15. exp Pamphlets/  16. exp Instructional Materials/  17. exp Standards/ or exp Guidelines/  18. exp Inservice Education/  19. exp Consultants/  20. exp Staff Development/  21. exp Continuing Education/  22. exp Professional Development/  23. (workshop* or seminar* or training or in-service).tw.  24. exp Program Development/  25. journal club.ti,ab.  26. (champion* adj change*).tw.  27. ((facilitat* or coordinat*) adj2 change*).tw.  28. or/14-27 | 29. exp Motivation/  30. exp Attitude Change/  31. exp Self Efficacy/  32. exp "Adoption (Ideas)"/  33. exp Information Dissemination/  34. ((research or evidence or guideline*) adj3 (implementation or utilization or utilisation or diffusion or translation)).tw.  35. ((predisposing or enabling or reinforcing) adj2 factor*).tw.  36. ((support* or impede*) adj change*).tw.  37. (behavi?or adj2 change*).tw.  38. (knowledge adj2 (utilization or utilisation or uptake or transfer* or implementation or dissemination or diffusion* or translation)).tw.  39. (increase* adj2 implementation).tw.  40. (implementation adj2 (program* or strategy or strategies)).tw.  41. exp Decision Making/  42. exp "Transfer of Training"/  43. or/29-42  44. and/13,28,43  45. limit 44 to (english language and yr="1985 -Current") |
| --- | --- |

**Database:** PASCAL

**Search name:** KT – allied professionals-3 | PASCAL 16Mar10

**Notes:** limits: english only, 1985-present

**Date searched:** 16Mar10

**Results:**

| KT - allied professionals-3  PASCAL - 16Mar10  1. occupational therap*.tw.  2. physiotherap*.tw.  3. physical therap*.tw.  4. (speech language adj (patholog* or therap*)).tw.  5. speech therap*.tw.  6. (dietitian* dietetics or dietician*).tw.  7. pharmacist*.mp. or pharmacy.tw. [mp=translated title, original title, abstract (english), heading word]  8. or/1-7  9. audiovisual aids.tw.  10. (pamphlets or brochures or handouts).tw.  11. consultant*.tw.  12. staff development.tw.  13. ((professional or clinical) adj competence).tw.  14. evidence-based*.tw.  15. (guideline adj2 adherence*).tw.  16. (adherence adj2 guideline*).tw.  17. (workshop* or seminar* or training or in-service).tw.  18. (champion* adj change*).tw.  19. (change adj2 agent*).tw.  20. ((facilitat* or coordinat*) adj2 change*).tw. | 21. motivation*.tw.  22. self efficacy.tw.  23. diffusion of innovation.tw.  24. ((research or evidence or guideline*) adj3 (implementation or utilization or utilisation or diffusion or translation)).tw.  25. ((predisposing or enabling or reinforcing) adj2 factor*).tw.  26. ((support* or impede*) adj change*).tw.  27. (behavi?or adj2 change*).tw.  28. (knowledge adj2 (utilization or utilisation or uptake or transfer* or implementation or dissemination or diffusion* or translation)).tw.  29. (increase* adj2 implementation).tw.  30. (implementation adj2 (program* or strategy or strategies)).tw.  31. decision making.tw.  32. or/21-31  33. 8 and 32  34. or/9-20  35. 8 and 34  36. 33 or 35  37. limit 36 to yr="1985 -Current" |
| --- | --- |

**Database:** EMBASE

**Search name:** KT – allied professionals-4 | EMBASE 17Mar10

**Notes:** limits: english only, 1985-present

**Date searched:** 17Mar10

**Results:**  207

| 1. exp physiotherapist/  2. exp physiotherapist assistant/  3. exp physiotherapist attitude/  4. exp occupational therapist/  5. exp occupational therapist attitude/  6. exp occupational therapy assistant/  7. exp speech language pathologist/  8. exp dietitian/  9. exp dietitian attitude/  10. exp pharmacist/  11. exp pharmacist attitude/  12. exp pharmacy technician/  13. or/1-12  14. exp practice guideline/  15. exp in service training/  16. exp reminder system/  17. exp clinical competence/  18. exp continuing education/  19. exp professional development/  20. exp professional standard/  21. (adherence adj3 guidelines).tw.  22. (guideline adj2 adherence).tw.  23. exp evidence based medicine/ or exp evidence based practice/  24. (workshop* or seminar* or training or in-service).tw. | 25. (champion* adj2 change*).tw.  26. (change adj2 agent*).tw.  27. ((facilitat* or coordinat*) adj2 change*).tw.  28. or/14-27  29. "diffusion of innovation".tw.  30. "organizational innovation".tw.  31. ((research or evidence or guideline*) adj3 (implementation or utilization or utilisation or diffusion or translation)).tw.  32. ((predisposing or enabling or reinforcing) adj2 factor*).tw.  33. ((support* or impede*) adj change*).tw.  34. (behavi?or adj2 change*).tw.  35. (knowledge adj2 (utilization or utilisation or uptake or transfer* or implementation or dissemination or diffusion* or translation)).tw.  36. (increase* adj2 implementation).tw.  37. (implementation adj2 (program* or strategy or strategies)).tw.  38. or/29-37  39. and/13,28,38  40. 13 and 38  41. 39 or 40  42. limit 41 to (english language and yr="1985 -Current") |
| --- | --- |

**Database:** IPA

**Search name:** KT – allied professionals-5 | IPA 17Mar10

**Notes:** limits: english only, 1985-present

**Date searched:** 17Mar10

**Results:**

| 1. physiotherapist*.tw.  2. occupational therapist*.mp. [mp=title, subject heading word, registry word, abstract, trade name/generic name]  3. ((occupational or physical) adj therap*).tw.  4. (speech adj2 patholog*).tw.  5. speech therapist*.tw.  6. speech language patholog*.tw.  7. (dietitian* or dietician*).tw.  8. (pharmacist* or pharmacy techn*).tw.  9. or/1-8  10. audiovisual aid*.tw.  11. pamphlet*.tw.  12. staff development.tw.  13. inservice training.tw.  14. reminder system*.tw.  15. continuing education.tw.  16. professional role.tw.  17. ((professional or clinical) adj2 competence).tw.  18. guideline adherence.tw.  19. (adherence adj3 guidelines).tw.  20. evidence based practice.tw.  21. evidence-based pharmac*.tw.  22. (workshop* or seminar* or training or in-service).tw. | 23. (champion* adj2 change*).tw.  24. (change adj2 agent*).tw.  25. ((facilitat* or coordinat*) adj2 change*).tw.  26. or/10-25  27. self efficacy.tw.  28. "organizational innovation".tw.  29. diffusion of innovation.tw.  30. ((research or evidence or guideline*) adj3 (implementation or utilization or utilisation or diffusion or translation)).tw.  31. ((predisposing or enabling or reinforcing) adj2  factor*).tw.  32. ((support* or impede*) adj change*).tw.  33. (behavi?or adj2 change*).tw.  34. (knowledge adj2 (utilization or utilisation or uptake or transfer* or implementation or dissemination or diffusion* or translation)).tw.  35. (increase* adj2 implementation).tw.  36. (implementation adj2 (program* or strategy or strategies)).tw.  37. or/27-36  38. 9 and 26 and 37  39. limit 38 to (english language and yr="1985 -Current") |
| --- | --- |

**Database:** Scopus

**Notes:** limits: english only, 1985-present

**Date searched:** 18Mar10

**Results:**  124

| (((((TITLE-ABS-KEY((occupational PRE/0 therapist*) OR (physical PRE/0 therapist*) OR (physiotherapist*) OR (speech W/2 pathologist*) OR (speech W/2 therapist*) OR (dietitian* OR dietician*) OR (diet* W/2 technician*) OR (diet W/2 technologist*) OR (pharmacist*) OR (pharm* W/2 technologist*) OR (pharm* W/2 technician*))) AND (TITLE-ABS-KEY((audiovisual) OR (pamphlet*) OR (practice PRE/0 guidelines) OR (in-service) OR (consultant*) OR (reminder*) OR (continuing PRE/0 education) OR (professional PRE/0 competence) OR (professional PRE/0 role) OR (guideline W/3 adherence) OR (evidence-based) OR (workshop*) OR (seminar*) OR (training) OR (journal PRE/1 club) OR (champion W/2 change*) OR (facilitat* W/2 change) OR (coordinat* W/2 change))) AND (TITLE-ABS-KEY((self PRE/0 efficacy) OR (organizational W/2 innovation) OR (diffusion W/2 innovation) OR (research W/3 implementation) (research W/3 utilization) OR (research W/3 diffusion) OR (research W/3 translation) OR (evidence W/3 implementation) OR (evidence W/3 utilization) OR (evidence W/3 diffusion) OR (evidence W/3 translation) OR (guideline* W/3 implementation) OR (guideline* utilization) OR (guideline* W/3 diffusion) OR (guideline W/3 translation) OR (support W/2 change*) OR (impede W/3 change*) OR (knowledge W/2 utilization) OR (knowledge W/2 uptake) OR (knowledge W/2 transfer*) OR (knowledge W/2 implementation) OR (knowledge W/2 dissemination) OR (knowledge W/2 diffusion) OR (knowledge W/2 translation) OR (implementation W/2 program*) OR (implementation W/2 strateg*) OR (increase W/2 implementation)))) OR ((TITLE-ABS-KEY((occupational PRE/0 therapist*) OR (physical PRE/0 therapist*) OR (physiotherapist*) OR (speech W/2 pathologist*) OR (speech W/2 therapist*) OR (dietitian* OR dietician*) OR (diet* W/2 technician*) OR (diet W/2 technologist*) OR (pharmacist*) OR (pharm* W/2 technologist*) OR (pharm* W/2 technician*))) AND (TITLE-ABS-KEY((self PRE/0 efficacy) OR (organizational W/2 innovation) OR (diffusion W/2 innovation) OR (research W/3 implementation) (research W/3 utilization) OR (research W/3 diffusion) OR (research W/3 translation) OR (evidence W/3 implementation) OR (evidence W/3 utilization) OR (evidence W/3 diffusion) OR (evidence W/3 translation) OR (guideline* W/3 implementation) OR (guideline* utilization) OR (guideline* W/3 diffusion) OR (guideline W/3 translation) OR (support W/2 change*) OR (impede W/3 change*) OR (knowledge W/2 utilization) OR (knowledge W/2 uptake) OR (knowledge W/2 transfer*) OR (knowledge W/2 implementation) OR (knowledge W/2 dissemination) OR (knowledge W/2 diffusion) OR (knowledge W/2 translation) OR (implementation W/2 program*) OR (implementation W/2 strateg*) OR (increase W/2 implementation))))) OR ((TITLE-ABS-KEY((occupational PRE/0 therap*) OR (physical PRE/0 therap*) OR (physiotherap*) OR (speech W/2 patholog*) OR (speech W/2 therap*) OR (dietitian* OR dietet*) OR (diet W/2 technolog*) OR (pharmac*) OR (pharm* W/2 techn*))) AND (TITLE-ABS-KEY((audiovisual) OR (pamphlet*) OR (practice PRE/0 guidelines) OR (in-service) OR (consultant*) OR (reminder*) OR (continuing PRE/0 education) OR (professional PRE/0 competence) OR (professional PRE/0 role) OR (guideline W/3 adherence) OR (evidence-based) OR (workshop*) OR (seminar*) OR (training) OR (journal PRE/1 club) OR (champion W/2 change*) OR (facilitat* W/2 change) OR (coordinat* W/2 change))) AND (TITLE-ABS-KEY((self PRE/0 efficacy) OR (organizational W/2 innovation) OR (diffusion W/2 innovation) OR (research W/3 implementation) (research W/3 utilization) OR (research W/3 diffusion) OR (research W/3 translation) OR (evidence W/3 implementation) OR (evidence W/3 utilization) OR (evidence W/3 diffusion) OR (evidence W/3 translation) OR (guideline* W/3 implementation) OR (guideline* utilization) OR (guideline* W/3 diffusion) OR (guideline W/3 translation) OR (support W/2 change*) OR (impede W/3 change*) OR (knowledge W/2 utilization) OR (knowledge W/2 uptake) OR (knowledge W/2 transfer*) OR (knowledge W/2 implementation) OR (knowledge W/2 dissemination) OR (knowledge W/2 diffusion) OR (knowledge W/2 translation) OR (implementation W/2 program*) OR (implementation W/2 strateg*) OR (increase W/2 implementation)))) OR ((TITLE-ABS-KEY((occupational PRE/0 therap*) OR (physical PRE/0 therap*) OR (physiotherap*) OR (speech W/2 patholog*) OR (speech W/2 therap*) OR (dietitian* OR dietet*) OR (diet W/2 technolog*) OR (pharmac*) OR (pharm* W/2 techn*))) AND (TITLE-ABS-KEY((self PRE/0 efficacy) OR (organizational W/2 innovation) OR (diffusion W/2 innovation) OR (research W/3 implementation) (research W/3 utilization) OR (research W/3 diffusion) OR (research W/3 translation) OR (evidence W/3 implementation) OR (evidence W/3 utilization) OR (evidence W/3 diffusion) OR (evidence W/3 translation) OR (guideline* W/3 implementation) OR (guideline* utilization) OR (guideline* W/3 diffusion) OR (guideline W/3 translation) OR (support W/2 change*) OR (impede W/3 change*) OR (knowledge W/2 utilization) OR (knowledge W/2 uptake) OR (knowledge W/2 transfer*) OR (knowledge W/2 implementation) OR (knowledge W/2 dissemination) OR (knowledge W/2 diffusion) OR (knowledge W/2 translation) OR (implementation W/2 program*) OR (implementation W/2 strateg*) OR (increase W/2 implementation))))) AND (PUBYEAR IS 1985 OR PUBYEAR AFT 1985) AND (LANGUAGE(english))) OR (TITLE(strategies for rehabilitation professionals to move evidence-based knowledge into practice: a systematic review)) |
| --- |

**Database:** CENTRAL

**Search name:** KT – allied professionals-6 | CENTRAL 17Mar10

**Notes:** limits: english only, 1985-present

**Date searched:** 17Mar10

**Results:**  61

| 1. exp Occupational Therapy/  2. (occupational adj therapist*).tw.  3. ((physical or occupational) adj2 therapy adj assistant).tw.  4. exp "Physical Therapy (Specialty)"/  5. (physical adj therapist*).tw.  6. physiotherapist*.tw.  7. exp Speech-Language Pathology/  8. (speech adj2 (therapist* or pathologist*)).tw.  9. exp dietetics/  10. (dietitian* or dietician*).tw.  11. (diet* adj2 techn*).tw.  12. exp Pharmacy/  13. Pharmacy Service, Hospital/  14. exp Pharmacists/  15. exp Pharmacists' Aides/  16. pharmacist*.tw.  17. (pharmacy adj (technologist* or technician*)).tw.  18. or/1-17  19. exp Audiovisual Aids/  20. exp Pamphlets/  21. education/  22. exp Teaching Materials/  23. exp Clinical Protocols/  24. exp practice guidelines as topic/  25. exp critical pathways/  26. exp inservice training/  27. exp consultants/  28. exp Staff Development/  29. exp Health Knowledge, Attitudes, Practice/  30. exp Reminder Systems/  31. exp Clinical Competence/  32. exp Education, Continuing/  33. professional development/  34. exp Professional Role/  63. (knowledge adj2 (utilization or utilisation or uptake or transfer* or implementation or dissemination or diffusion* or translation)).tw.  64. (increase adj2 implementation).tw.  65. (implementation adj2 (program or strategy or strategies)).tw. | 35. exp Professional Competence/  36. exp Guideline Adherence/  37. (adherence adj3 guidelines).tw.  38. (guideline adj3 adherence).tw.  39. exp "Attitude of Health Personnel"/  40. exp Evidence-Based Practice/  41. exp evidence-based medicine/  42. (workshop* or seminar* or training or in-service).tw.  43. exp "Process Assessment (Health Care)"/  44. exp "Outcome and Process Assessment (Health Care)"/  45. exp "Delivery of Health Care"/  46. exp program development/mt  47. exp Health Plan Implementation/mt, og [Methods, Organization & Administration]  48. (journal adj club).ti,ab.  49. exp Quality Assurance, Health Care/  50. organizational case studies/  51. (champion* adj2 change*).tw.  52. (change adj2 agent*).tw.  53. ((facilitat* or coordinat*) adj2 change*).tw.  54. or/19-53  55. exp motivation/  56. exp Self Efficacy/  57. exp Organizational Innovation/  58. exp "diffusion of innovation"/  59. ((research or evidence or guideline*) adj3 (implementation or utilization or utilisation or diffusion or translation)).tw.  60. ((predisposing or enabling or reinforcing) adj2 factor*).tw.  61. ((support or impede) adj2 change*).tw.  62. (behavio?r adj2 change*).tw.  66. decision making/  67. or/55-66  68. and/18,54,67  69. limit 68 to (english language and yr="1985 -Current") |
| --- | --- |

RESULTS SUMMARY:

| **Database** | **Dates searched** | **Date search ran** | **Number of results** |
| --- | --- | --- | --- |
| MEDLINE (OVID) | 1985-current | 16Mar10 | 1004 |
| CINAHL (EBSCO) | 1985-current | 17Mar10 | 973 |
| Scopus (Elsevier) | 1985-current | 18Mar10 | 124 |
| ERIC (OVID) | 1985-current | 16Mar10 | 80 |
| PASCAL (OVID) | 1985-current | 16Mar10 | 164 |
| IPA (OVID) | 1985-current | 17Mar10 | 207 |
| EMBASE (OVID) | 1985-current | 17Mar10 | 183 |
| CENTRAL (OVID) | 1985-current | 18Mar10 | 61 |
| **TOTAL RESULTS (with duplicates)** | | | **2796** |

**Duplication Delete rule:**

**MEDLINE 🡪CINAHL 🡪EMBASE🡪SCOPUS🡪CENTRAL🡪IPA🡪ERIC🡪PASCAL**
