# Supplementary material for: Systematic review of knowledge translation strategies in the allied health professions
Source: Implement Sci. 2012 Jul 25;7:70. doi: 10.1186/1748-5908-7-70 (PMC3780719; doi:10.1186/1748-5908-7-70)
Supplement: Additional file 2 — Cochrane Effective Practice and Organisation of Care Review Group. [file 1748-5908-7-70-S2.pdf]

# Cochrane Effective Practice and Organisation of Care Review Group

# DATA COLLECTION CHECKLIST

# Cochrane Effective Practice and Organisation of Care Review Group (EPOC)

## Data Collection Checklist

**CONTENTS**


| Item | | Page |
|---|---|---|
| | **Introduction** | 5-6 |
| 1 | **Inclusion criteria*** | 7-8 |
| 1.1 | Study design* | 7 |
| 1.1.1 | Randomised controlled trial* | |
| 1.1.2 | Controlled clinical trial* | |
| 1.1.3 | Controlled before and after study* | |
| 1.1.4 | Interrupted time series* | |
| 1.2 | Methodological inclusion criteria* | 8 |
| 2 | **Interventions*** | 9-12 |
| 2.1 | Type of intervention | 9 |
| 2.1.1 | Professional interventions* | 9 |
| 2.1.2 | Financial interventions* | 10 |
| 2.1.2.1 | Provider interventions* | |
| 2.1.2.2 | Patient interventions* | |
| 2.1.3 | Organisational interventions* | 11 |
| 2.1.3.1 | Provider orientated interventions* | |
| 2.1.3.2 | Patient orientated interventions* | |
| 2.1.3.3 | Structural interventions* | |
| 2.1.4 | Regulatory interventions* | 12 |
| 2.2 | **Controls*** | 13 |
| 3 | **Type of targeted behaviour*** | 13 |
| 4 | **Participants*** | 14-15 |
| 4.1 | Characteristics of participating providers* | 14 |
| 4.1.1 | Profession* | |

| Item | Page |
|---|---|

| | | |
|---|---|---|
| 4.1.2 | Level of training* | |
| 4.1.3 | Clinical speciality* | |
| 4.1.4 | Age | |
| 4.1.5 | Time since graduation | |
| 4.2 | Characteristics of participating patients* | 15 |
| 4.2.1 | Clinical problem* | |
| 4.2.2 | Other patient characteristics | |
| 4.2.3 | Number of patients included in the study* | |
| 5 | **Setting*** | 16 |
| 5.1 | Reimbursement system | |
| 5.2 | Location of care* | |
| 5.3 | Academic Status* | |
| 5.4 | Country* | |
| 5.5 | Proportion of eligible providers from the sampling frame* | |
| 6 | **Methods*** | 17 |
| 6.1 | Unit of allocation* | |
| 6.2 | Unit of analysis* | |
| 6.3 | Power calculation* | |
| 6.4 | Quality criteria* | 17-22 |
| 6.4.1 | Quality criteria for randomised controlled trials (RCT) and controlled clinical trials (CCT)* | 17 |
| 6.4.2 | Quality criteria for controlled before and after (CBA) designs* | 19 |
| 6.4.3 | Quality criteria for interrupted time series (ITS) designs* | 20 |
| 6.4.4 | Consumer involvement* | 22 |
| 7 | **Prospective identification by investigators of barriers to change** | 22 |
| 8 | **Intervention*** | 23-25 |
| 8.1 | Characteristics of the intervention* | 23 |

| Item | | Page |
|---|---|---|
| 8.2 | Nature of desired change | |
| 8.3 | Format | |
| 8.4 | Source | 24 |
| 8.5 | Intervention based upon implementation of clinical practice guidelines | |
| 8.6 | Clinical practice guidelines developed through formal consensus process | |
| 8.7 | Recipient | |
| 8.8 | Deliverer | |
| 8.9 | Timing | 25 |
| 8.10 | Setting of intervention | |
| 8.11 | Source of funding | |
| 8.12 | Ethical approval | |
| **9** | **Outcomes*** | 26 |
| 9.1 | Description of the main outcome measure(s)* | |
| 9.2 | Length of time during which outcomes were measured after initiation of the intervention* | |
| 9.3 | Length of post-intervention follow-up period* | |
| 9.4 | Possible ceiling effect* | |
| **10** | **Results*** | 27-28 |
| 10.1 | Randomised controlled trials and controlled clinical trials* | |
| 10.2 | Controlled before and after designs* | |
| 10.3 | Interrupted time series* | |


For items marked with *, please see introduction on page 5.

# Cochrane Effective Practice and Organisation of Care Review Group (EPOC)

Cochrane Effective Practice and Organisation of Care Group (EPOC)
Institute of Population Health, University of Ottawa
1 Stewart St.
Ottawa, Ontario, Canada K1N 6N5

Alain Mayhew, Review Group Co-ordinator
Tel: (613) 562-5800 ext 2361
Fax: (613)562-5659
E-mail: epoc@uottawa.ca or al.mayhew@uottawa.ca

## *INTRODUCTION*

The purpose of the data extraction checklist is to provide a guide to reviewers about the type of relevant information that could be extracted from primary studies. Each review is different and reviewers will need to adapt the checklist to suit their purposes. In order to make reviews useful to readers, certain types of information (for example quality assessments) should be available in all reviews. These standard items are marked with an asterisk (*).

## METHODS

Once relevant studies have been identified for possible inclusion in a review, data regarding inclusion criteria (design, participants, interventions, and outcomes), quality criteria and results should be extracted independently by two reviewers. Other data can be extracted by one reviewer and checked by another. Reviewers should consider how data will be presented in the appropriate software, Review Manager (RevMan). Examples of the Table of Included Studies, and Results Tables are available from the editorial office. These examples illustrate the standard format used by EPOC.

**Prior to entering data into RevMan, reviewers should check with their assigned contact editor**.

### Paper or electronic forms

Reviewers may choose from a number of different options to record data extraction, including the EPOC 'data collection template' (available in paper and electronic format) and an Idealist database (available from the Blackwell publishing group), incorporating the group's 'register' definition file which is available from the editorial office. Choice of format for data collection will depend upon strategies used for checking data. Reviewers should be aware that data tables created using word processing software cannot be readily transferred into RevMan (at present). If reviewers enter data directly into RevMan, then reviews should be exported frequently for safekeeping.

During data collection, it may be useful for reviewers to indicate the source page numbers against each item recorded as this facilitates later comparisons of extracted data.

Discrepancies between reviewers should be resolved by discussion and any decisions that cannot be resolved easily should be referred to the contact editor for the review.

Data that is missing or ‘not clear’ in a published report should be marked clearly on the data collection form.  Missing information should be sought from the corresponding author of a paper.

# 1. INCLUSION CRITERIA

The items 1.1 - 1.2 (inclusive) in this section determine whether a study should be included in an EPOC review.

## 1.1 Study design

The design of the study is (state which):

1.1.1 *Randomised controlled trial (RCT)* i.e. a trial in which the participants (or other units) were definitely assigned prospectively to one or two (or more) alternative forms of health care using a process of random allocation (e.g. random number generation, coin flips).

1.1.2 *Controlled clinical trial (CCT)* may be a trial in which participants (or other units) were:

a) definitely assigned prospectively to one or two (or more) alternative forms of health care using a quasi-random allocation method (e.g. alternation, date of birth, patient identifier) or;

b) possibly assigned prospectively to one or two (or more) alternative forms of health care using a process of random or quasi-random allocation.

1.1.3 *Controlled before and after study (CBA)* i.e. involvement of intervention and control groups other than by random process, and inclusion of baseline period of assessment of main outcomes. There are three minimum criteria for inclusion of CBAs in EPOC reviews:

a) *Contemporaneous data collection*
Score DONE pre and post intervention periods for study and control sites are the same.
Score NOT CLEAR if it is not clear in the paper, e.g. dates of collection are not mentioned in the text. (N.B. the paper should be discussed with the contact editor for the review before data extraction is undertaken).
Score NOT DONE if data collection was not conducted contemporaneously during pre and post intervention periods for study and control sites.

b) *Appropriate choice of control site:*
Studies using second site as controls:
Score DONE if study and control sites are comparable with respect to dominant reimbursement system, level of care, setting of care and academic status.
Score NOT CLEAR if not clear from paper whether study and control sites are comparable. (N.B. the paper should be discussed with the contact editor for the review before data extraction is undertaken).
Score NOT DONE if study and control sites are not comparable.

c) Minimum number of sites:
Score DONE if there are a minimum of two intervention sites and two control sites.
Score NOT DONE if there are less than two intervention sites and two control sites.

1.1.4 *Interrupted time series (ITS)* i.e. a change in trend attributable to the intervention. There are two minimum criteria for inclusion of ITS designs in EPOC reviews:

a) *Clearly defined point in time when the intervention occurred.*
Score DONE if reported that intervention occurred at a clearly defined point in time.
Score NOT CLEAR if not reported in the paper (will be treated as NOT DONE if information cannot be obtained from the authors).

Score NOT DONE if reported that intervention did not occur at a clearly defined point in time.

b) *At least three data points before and three after the intervention.*
Score DONE if 3 or more data points before and 3 or more data points recorded after the intervention.
Score NOT CLEAR if not specified in paper e.g. number of discrete data points not mentioned in text or tables (will be treated as NOT DONE if information cannot be obtained from the authors).
Score NOT DONE if less than 3 data points recorded before and 3 data points recorded after intervention.

***If the study is not any of the above designs, it should not be included in an EPOC review. If you scored NOT DONE for any of the above criteria in item 1.1, the study should not be included in an EPOC review. If reviewers are unsure of the study design, the paper should be discussed with the contact editor for the review before data extraction is undertaken.***

## 1.2 Methodological inclusion criteria

The minimum methodological inclusion criteria across all study designs are:

a) *The objective measurement of performance/provider behaviour of health/patient outcome(s) in a clinical* ***not test*** *situation.*
Score DONE (e.g. drug levels assessed by a test, performance of providers against pre-set criteria, number of tests ordered, diastolic blood pressure, number of caesarean sections performed etc.). Outcome measures such as provider satisfaction with work or patient satisfaction with care may be included if they are assessed using a questionnaire with known reliability and validity.
Score NOT CLEAR (the paper should be discussed with the contact editor for the review before data extraction is undertaken).
Score NOT DONE (e.g. self-reported data, measurement of attitudes, beliefs, perceptions or satisfaction).

b) *Relevant and interpretable data presented or obtainable.*
Score DONE if data was presented or obtainable.
Score NOT CLEAR (the paper should be discussed with the contact editor for the review before data extraction is undertaken).
Score NOT DONE if relevant data was not presented and is clearly unobtainable.

***If either of the above criteria in item 1.2 is scored as NOT DONE, the study should not be included in an EPOC review.***

# 2. INTERVENTIONS

EPOC reviews include professional, financial, organisational or regulatory interventions.

State all interventions for each comparison/study group. (The categories are not mutually exclusive.)

## 2.1 Type of intervention

### 2.1.1 *Professional interventions*

a) Distribution of educational materials (Distribution of published or printed recommendations for clinical care, including clinical practice guidelines, audio-visual materials and electronic publications. The materials may have been delivered personally or through mass mailings.)

b) Educational meetings (Health care providers who have participated in conferences, lectures, workshops or traineeships.)

c) Local consensus processes (Inclusion of participating providers in discussion to ensure that they agreed that the chosen clinical problem was important and the approach to managing the problem was appropriate.)

d) Educational outreach visits (Use of a trained person who met with providers in their practice settings to give information with the intent of changing the provider's practice. The information given may have included feedback on the performance of the provider(s).

e) Local opinion leaders (Use of providers nominated by their colleagues as 'educationally influential'. The investigators must have explicitly stated that their colleagues identified the opinion leaders.)

f) Patient mediated interventions (New clinical information (not previously available) collected directly from patients and given to the provider e.g. depression scores from an instrument.)

g) Audit and feedback (Any summary of clinical performance of health care over a specified period of time. The summary may also have included recommendations for clinical action. The information may have been obtained from medical records, computerised databases, or observations from patients.)

**The following interventions are excluded:**

- Provision of new clinical information not directly reflecting provider performance which was collected from patients e.g. scores on a depression instrument, abnormal test results. These interventions should be described as patient mediated.
- Feedback of individual patients' health record information in an alternate format (e.g. computerised). These interventions should be described as organisational.

h) Reminders (Patient or encounter specific information, provided verbally, on paper or on a computer screen, which is designed or intended to prompt a health professional to recall information. This would usually be encountered through their general education; in the medical records or through interactions with peers, and so remind them to perform or avoid

some action to aid individual patient care. Computer aided decision support and drugs dosage are included.)

i) Marketing (Use of personal interviewing, group discussion (‘focus groups’), or a survey of targeted providers to identify barriers to change and subsequent design of an intervention that addresses identified barriers.)

j) Mass media ((i) varied use of communication that reached great numbers of people including television, radio, newspapers, posters, leaflets, and booklets, alone or in conjunction with other interventions; (ii) targeted at the population level.)

k) Other (Other categories to be agreed in consultation with the EPOC editorial team.)

### 2.1.2 *Financial interventions*

#### 2.1.2.1 *Provider interventions*

a) Fee-for-service (provider has been paid for number and type of service delivered)

b) Prepaid (no other description)

c) Capitation (provider was paid a set amount per patient for providing specific care)

d) Provider salaried service (provider received basic salary for providing specific care)

e) Prospective payment (provider was paid a fixed amount for health care in advance)

f) Provider incentives (provider received direct or indirect financial reward or benefit for doing specific action)

g) Institution incentives (institution or group of providers received direct or indirect financial rewards or benefits for doing specific action)

h) Provider grant/allowance (provider received direct or indirect financial reward or benefit not tied to specific action)

i) Institution grant/allowance (institution or group of providers received direct or indirect financial reward or benefit not tied to specific action)

j) Provider penalty (provider received direct or indirect financial penalty for inappropriate behaviour)

k) Institution penalty (institution or group of providers received direct or indirect financial penalty for inappropriate behaviour)

l) Formulary (added or removed from reimbursable available products)

m) Other (other categories to be agreed in consultation with the EPOC editorial team)

#### 2.1.2.2 *Patient interventions*

a) Premium (Patient payment for health insurance. It is important to determine if the patient paid the entire premium, or if the patient's employer paid some of it. This includes different types of insurance plans.)

b) Co-payment (Patient payment at the time of health care delivery in addition to health insurance e.g. in many insurance plans that cover prescription medications the patient may pay 5 dollars per prescription, with the rest covered by insurance.)

c) User-fee (Patient payment at the time of health care delivery.)

d) Patient incentives (Patient received direct or indirect financial reward or benefit for doing or encouraging them to do specific action.)

e) Patient grant/allowance (Patient received direct or indirect financial reward or benefit not tied to specific action.)

f) Patient penalty (Patient received direct or indirect financial penalty for specified behaviour e.g. reimbursement limits on prescriptions.)

g) Other (other categories to be agreed in consultation with the EPOC editorial team)

### 2.1.3 *Organisational interventions*

#### 2.1.3.1 *Provider orientated interventions*

a) Revision of professional roles (Also known as 'professional substitution', 'boundary encroachment' and includes the shifting of roles among health professionals. For example, nurse midwives providing obstetrical care; pharmacists providing drug counselling that was formerly provided by nurses and physicians; nutritionists providing nursing care; physical therapists providing nursing care. Also includes expansion of role to include new tasks.)

b) Clinical multidisciplinary teams (creation of a new team of health professionals of different disciplines or additions of new members to the team who work together to care for patients)

c) Formal integration of services (bringing together of services across sectors or teams or the organisation of services to bring all services together at one time also sometimes called 'seamless care')

d) Skill mix changes (changes in numbers, types or qualifications of staff)

e) Continuity of care (including one or many episodes of care for inpatients or outpatients)
- Arrangements for follow-up.
- Case management (including co-ordination of assessment, treatment and arrangement for referrals)

f) Satisfaction of providers with the conditions of work and the material and psychic rewards (e.g. interventions to 'boost morale')

g) Communication and case discussion between distant health professionals (e.g. telephone links; telemedicine; there is a television/video link between specialist and remote nurse practitioners)

h) Other (other categories to be agreed in consultation with the EPOC editorial team)

2.1.3.2 *Patient orientated interventions*

a) Mail order pharmacies (e.g. compared to traditional pharmacies)

b) Presence and functioning of adequate mechanisms for dealing with patients' suggestions and complaints

c) Consumer participation in governance of health care organisation

d) Other (other categories to be agreed in consultation with the EPOC editorial team)

2.1.3.3 *Structural interventions*

a) Changes to the setting/site of service delivery (e.g. moving a family planning service from a hospital to a school)

b) Changes in physical structure, facilities and equipment (e.g change of location of nursing stations, inclusion of equipment where technology in question is used in a wide range of problems and is not disease specific, for example an MRI scanner.)

c) Changes in medical records systems (e.g. changing from paper to computerised records, patient tracking systems)

d) Changes in scope and nature of benefits and services

e) Presence and organisation of quality monitoring mechanisms

f) Ownership, accreditation, and affiliation status of hospitals and other facilities

g) Staff organisation

h) Other (other categories to be agreed in consultation with the EPOC editorial team)

2.1.4 *Regulatory interventions*

Any intervention that aims to change health services delivery or costs by regulation or law. (These interventions may overlap with organisational and financial interventions.)

a) Changes in medical liability

b) Management of patient complaints

c) Peer review

d) Licensure

e) Other (other categories to be agreed in consultation with the EPOC editorial team)

## 2.2 Controls

The study used was (specify):

a) No intervention control group

b) Standard practice control group (if different to (a) above)

c) Untargeted activity

d) Other (e.g. another intervention)

## 3. TYPE OF TARGETED BEHAVIOUR (state more than one where appropriate)

a) Clinical prevention services

b) Diagnosis

c) Test ordering

d) Referrals

e) Procedures

f) Prescribing

g) General management of a problem (e.g. the treatment of hypertension)

h) Patient education/advice

i) Professional-patient communication

j) Record keeping

k) Financial (resource use)

l) Discharge planning

m) Patient outcome

n) Other (specify)

o) NOT CLEAR

# 4. PARTICIPANTS

## 4.1 Characteristics of participating providers

4.1.1 ***Profession***

a) Physicians

b) Nurses

c) Pharmacists

d) Physiotherapists

e) Dentists

f) Psychologists

g) Mixed (specify)

h) Other provider (specify)

i) NOT CLEAR

4.1.2 ***Level of training***

a) In training (practising under supervision)

b) Accredited/licensed (i.e. fully trained, able to practice without supervision)

c) Mixed

d) NOT CLEAR

4.1.3 ***Clinical speciality*** *(list all as appropriate)*

a) General/family practice

b) Internal medicine

c) Surgery

d) Psychiatry

e) Paediatrics

f) Obstetrics and gynaecology

g) Laboratory medicine

h) Radiology

i) Other (specify)

j) Not applicable

k) NOT CLEAR

4.1.4 *<u>Age</u>*

State the mean age of participating providers (score NOT CLEAR if information is not available)

4.1.5 *<u>Time since graduation</u> (or years in practice)*

Score NOT CLEAR if information is not available.

## 4.2 **Characteristics of participating patients**

4.2.1 *<u>Clinical problem</u>*

State the area(s) that the intervention targets (e.g. hypertension, oncology, preventive services etc). (Score NOT CLEAR if information is not available.)

4.2.2 *<u>Other patient characteristics</u> (for each, score NOT CLEAR if information not available)*

a) Age

b) Gender

c) Ethnicity

d) Other (specify)

4.2.3 *<u>Number of patients included in the study</u> (e.g. those who entered the study) (for each, score NOT CLEAR if information not available)*

a) Episodes of care

b) Patients

c) Providers

d) Practices

e) Hospitals

f) Communities or regions

# 5. SETTING

## 5.1 Reimbursement system

a) Fee for service (provider was paid for the number and type of services delivered)

b) Capitation (provider was paid a set amount per patient for providing specific care)

c) Prospective payment

d) Global budget

e) Mixed

f) Other (specify)

g) NOT CLEAR

## 5.2 Location of care

a) Inpatient care

b) Outpatient care (e.g. ambulatory care provided by specialists/hospitals)

c) Community based care

d) Mixed

e) NOT CLEAR

## 5.3 Academic status

a) University based/teaching setting (i.e. not simply university affiliation)

b) Non-teaching setting

c) Mixed

d) NOT CLEAR

## 5.4 Country

Score NOT CLEAR if information is not available.

## 5.5 Proportion of eligible providers (or allocation units)

Proportion of eligible providers (or allocation units) who participated in the evaluation out of the total number in the sampling frame (state/calculate the percentage of providers in the target population who were allocated to study groups). (Score NOT CLEAR if information is not available.)

# 6. METHODS

## 6.1 Unit of allocation (i.e. who or what was allocated to study groups)

a) Patient

b) Provider

c) Practice

d) Institution

e) Community

f) Firm

g) Clinic day

h) Other (specify)

i) NOT CLEAR

## 6.2 Unit of analysis (i.e. results analysed as events per practice)

a) Patient

b) Provider

c) Practice

d) Institution

e) Community

f) Firm

g) Clinic day

h) Other (specify)

i) NOT CLEAR

## 6.3 Power calculation

Score DONE if study has sufficient statistical power to detect clinically important effects as statistically significant and record power.
Score NOT CLEAR if not reported.
Score NOT DONE if authors specifically report that the study was under-powered

## 6.4 Quality criteria

### 6.4.1 *Quality criteria for randomised controlled trials (RCTs & CCTs)*

(N.B. See 6.4.2 and 6.4.3 for quality criteria for CBA and ITS respectively.)

Seven standard criteria are used for randomised controlled trials and controlled clinical trials included in EPOC reviews:

a) Concealment of allocation (protection against selection bias)
Score DONE if
- the unit of allocation was by institution, team or professional and any random process is described explicitly, e.g. the use of random number tables or coin flips;
- the unit of allocation was by patient or episode of care and there was some form of centralised randomisation scheme, an on-site computer system or sealed opaque envelopes were used.

Score NOT CLEAR if
- the unit of allocation is not described explicitly;
- the unit of allocation was by patient or episode of care and the authors report using a 'list' or 'table', 'envelopes' or 'sealed envelopes' for allocation.

Score NOT DONE if
- the authors report using alternation such as reference to case record numbers, dates of birth, day of the week or any other such approach (as in CCTs);
- the unit of allocation was by patient or episode of care and the authors report using any allocation process that is entirely transparent before assignment such as an open list of random numbers or assignments;
- allocation was altered (by investigators, professionals or patients).

b) Follow-up of professionals (protection against exclusion bias)
Score DONE if outcome measures obtained for 80-100% of subjects randomised. (Do not assume 100% follow up unless stated explicitly.);
Score NOT CLEAR if not specified in the paper;
Score NOT DONE if outcome measures obtained for less than 80% of subjects randomised.

c) Follow-up of patients or episodes of care
Score DONE if outcome measures obtained for 80-100% of subjects randomised or for patients who entered the trial. (Do not assume 100% follow up unless stated explicitly.) Score DONE if there is an objective data collection system;
Score NOT CLEAR if not specified in the paper;
Score NOT DONE if outcome measures obtained for less than 80% of subjects randomised or for less than 80% of patients who entered the trial.

d) Blinded assessment of primary outcome(s)* (protection against detection bias)
Score DONE if the authors state explicitly that the primary outcome variables were assessed blindly OR the outcome variables are objective, e.g. length of hospital stay, drug levels as assessed by a standardised test;
Score NOT CLEAR if not specified in the paper;
Score NOT DONE if the outcome(s) were not assessed blindly.

***Primary outcome(s) are those variables that correspond to the primary hypothesis or question as defined by the authors. In the event that some of the primary outcome variables were assessed in a blind fashion and others were not, score each separately and label each outcome variable clearly.***

e) Baseline measurement
Score DONE if performance or patient outcomes were measured prior to the intervention, and no substantial differences were present across study groups;

Score NOT CLEAR if baseline measures are not reported, or if it is unclear whether baseline measures are substantially different across study groups;
Score NOT DONE if there are differences at baseline in main outcome measures likely to undermine the post intervention differences (e.g. are differences between the groups before the intervention similar to those found post intervention).

f) Reliable primary outcome measure(s)*
Score DONE if two or more raters with at least 90% agreement or kappa greater than or equal to 0.8 OR the outcome is obtained from some automated system e.g. length of hospital stay, drug levels as assessed by a standardised test;
Score NOT CLEAR if reliability is not reported for outcome measures that are obtained by chart extraction or collected by an individual;
Score NOT DONE if agreement is less than 90% or kappa is less than 0.8.

***In the event that some outcome variables were assessed in a reliable fashion and others were not, score each separately on the back of the form and label each outcome variable clearly.***

g) Protection against contamination
Score DONE if allocation was by community, institution or practice and it is unlikely that the control received the intervention;
Score NOT CLEAR if professionals were allocated within a clinic or practice and it is possible that communication between experimental and group professionals could have occurred;
Score NOT DONE if it is likely that the control group received the intervention (e.g. cross-over trials or if patients rather than professionals were randomised).

### 6.4.2 Quality criteria for controlled before and after (CBA) designs

Seven standard criteria are used for CBAs included in EPOC reviews:

a) Baseline measurement
Score DONE if performance or patient outcomes were measured prior to the intervention, and no substantial differences were present across study groups (e.g. where multiple pre intervention measures describe similar trends in intervention and control groups);
Score NOT CLEAR if baseline measures are not reported, or if it is unclear whether baseline measures are substantially different across study groups;
Score NOT DONE if there are differences at baseline in main outcome measures likely to undermine the post intervention differences (e.g. are differences between the groups before the intervention similar to those found post intervention).

b) Characteristics for studies using second site as control
Score DONE if characteristics of study and control providers are reported and similar;
Score NOT CLEAR if it is not clear in the paper e.g. characteristics are mentioned in the text but no data are presented;
Score NOT DONE if there is no report of characteristics either in the text or a table OR if baseline characteristics are reported and there are differences between study and control providers.

c) Blinded assessment of primary outcome(s)* (protection against detection bias)

Score DONE if the authors state explicitly that the primary outcome variables were assessed blindly OR the outcome variables are objective e.g. length of hospital stay, drug levels as assessed by a standardised test;
Score NOT CLEAR if not specified in the paper;
Score NOT DONE if the outcomes were not assessed blindly.

***Primary outcome(s) are those variables that correspond to the primary hypothesis or question as defined by the authors. In the event that some of the primary outcome variables were assessed in a blind fashion and others were not, score each separately and label each outcome variable clearly.***

d) Protection against contamination
Studies using second site as control
Score DONE if allocation was by community, institution, or practice and is unlikely that the control group received the intervention;
Score NOT CLEAR if providers were allocated within a clinic or practice and communication between experimental and group providers was likely to occur;
Score NOT DONE if it is likely that the control group received the intervention (e.g. cross-over studies or if patients rather than providers were randomised).

e) Reliable primary outcome measure(s)
Score DONE if two or more raters with at least 90% agreement or kappa greater than or equal to 0.8 OR the outcome is obtained from some automated system e.g. length of hospital stay, drug levels as assessed by a standardised test;
Score NOT CLEAR if reliability is not reported for outcome measures that are obtained by chart extraction or collected by an individual;
Score NOT DONE if agreement is less than 90% or kappa is less than 0.8.

***In the event that some outcome variables were assessed in a reliable fashion and others were not, score each separately and label each outcome variable clearly.***

f) Follow-up of professionals (protection against exclusion bias)
Score DONE if outcome measures obtained 80-100% subjects allocated to groups. (Do not assume 100% follow-up unless stated explicitly.);
Score NOT CLEAR if not specified in the paper;
Score NOT DONE if outcome measures obtained for less than 80% of patients allocated to groups.

g) Follow-up of patients
Score DONE if outcome measures obtained 80-100% of patients allocated to groups or for patients who entered the study. (Do not assume 100% follow-up unless stated explicitly.);
Score NOT CLEAR if not specified in the paper;
Score NOT DONE if outcome measures obtained for less than 80% of patients allocated to groups or for less than 80% of patients who entered the study.

### 6.4.3 *Quality criteria for interrupted time series (ITSs)*

The following seven standard criteria should be used to assess the methodology quality of ITS designs included in EPOC reviews. Each criterion is scored DONE,

NOT CLEAR or NOT DONE. The results of the quality assessment for each study are reported in the Table of Included Studies in RevMan. Examples can be obtained from the EPOC review group co-ordinator.

a) Protection against secular changes

- The intervention is independent of other changes.
  Score DONE if the intervention occurred independently of other changes over time;
  Score NOT CLEAR if not specified (will be treated as NOT DONE if information cannot be obtained from the authors);
  Score NOT DONE if reported that intervention was not independent of other changes in time.

b) Data were analysed appropriately

Score DONE if ARIMA models were used **OR** time series regression models were used to analyse the data and serial correlation was adjusted/tested for;
Score NOT CLEAR if not specified (will be treated as NOT DONE if information cannot be obtained from the authors);
Score NOT DONE if it is clear that neither of the conditions above not met.

c) Reason for the number of points pre and post intervention given

Score DONE if rationale for the number of points stated (eg monthly data for 12 months post-intervention was used because the anticipated effect was expected to decay) **OR** sample size calculation performed;
Score NOT CLEAR if not specified (will be treated as NOT DONE if information cannot be obtained from the authors);
Score NOT DONE if it is clear that neither of the conditions above met.

d) Shape of the intervention effect was specified

Score DONE if a rational explanation for the shape of intervention effect was given by the author(s);
Score NOT CLEAR if not specified (will be treated as NOT DONE if information cannot be obtained from the authors);
Score NOT DONE if it is clear that the condition above is not met

e) Protection against detection bias

- Intervention unlikely to affect data collection
  Score DONE if reported that intervention itself was unlikely to affect data collection (for example, sources and methods of data collection were the same before and after the intervention);
  Score NOT CLEAR if not reported (will be treated as NOT DONE if information cannot be obtained from the authors);
  Score NOT DONE if the intervention itself was likely to affect data collection (for example, any change in source or method of data collection reported).

- Blinded assessment of primary outcome(s)*
  Score DONE if the authors state explicitly that the primary outcome variables were assessed blindly OR the outcome variables are objective e.g. length of hospital stay, drug levels as assessed by a standardised test;
  Score NOT CLEAR if not specified (will be treated as NOT DONE if information cannot be obtained from the authors);
  Score NOT DONE if the outcomes were not assessed blindly.

***Primary outcome(s) are those variables that correspond to the primary hypothesis or question as defined by the authors. In the event that some of the primary outcome variables were assessed in a blind fashion and others were not, score each separately and label each outcome variable clearly.***

c) Completeness of data set
Score DONE if data set covers 80-100% of total number of participants or episodes of care in the study;
Score NOT CLEAR if not specified (will be treated as NOT DONE if information cannot be obtained from the authors);
Score NOT DONE if data set covers less than 80% of the total number of participants or episodes of care in the study.

d) Reliable primary outcome measure(s)*
Score DONE if two or more raters with at least 90% agreement or kappa greater than or equal to 0.8 OR the outcome is obtained from some automated system e.g. length of hospital stay, drug levels as assessed by a standardised test;
Score NOT CLEAR if reliability is not reported for outcome measures that are obtained by chart extraction or collected by an individual (will be treated as NOT DONE if information cannot be obtained from the authors);
Score NOT DONE if agreement is less than 90% or kappa is less than 0.8.

***In the event that some outcome variables were assessed in a reliable fashion and others were not, score each separately.***

6.4.4 <u>*Consumer involvement*</u>

Were consumers (i.e. potential patients) involved at any point of the design, conduct or interpretation of the study? (E.g., consumers involved in clinical practice guideline development, or their views collected.)

Score DONE if specified in paper, and give details;
Score NOT CLEAR if not reported;
Score NOT DONE if consumers explicitly not involved.

## 7. PROSPECTIVE IDENTIFICATION BY INVESTIGATORS OF BARRIERS TO CHANGE

Investigators identified specific barriers to change in the target population, which were addressed by the intervention

a) Information management

b) Clinical uncertainty

c) Sense of competence

d) Perceptions of liability

e) Patient expectations

f) Standards of practice

g) Financial disincentives

h) Administrative constraints

i) Other (please specify)

j) NOT DONE

k) NOT CLEAR

# 8. INTERVENTION

## 8.1 Characteristics of the intervention

a) Evidence base of recommendation.
Score DONE if recommendations appear to be based on good evidence (e.g. there is clear reference to a systematic review or at least one randomised controlled trial);
Score NOT CLEAR if not specified in the paper;
Score NOT DONE if explicitly not evidence based.

b) Purpose of recommendations
- Appropriate management.
- Cost containment.
- Other (specify).
- NOT CLEAR

## 8.2 Nature of desired change

a) Initiation of new management (i.e. the introduction of a new technology)

b) Stopping introduction of new management

c) Reduction of established management

d) Increase established management

e) Cessation of established management

f) Modification of established management (e.g. increased management in one activity, reduction in another)

g) NOT CLEAR

## 8.3 Format

For each intervention state the medium employed

a) Interpersonal

b) Paper

c) Audio/visual

d) Computer/interactive

e) Multiple media used

f) Other (specify)

g) NOT CLEAR

8.4 **Source**

a) Local clinicians

b) Local expert body

c) National professional expert body

d) National government expert body

e) International professional expert body

f) International government expert body

g) Other (specify)

h) NOT CLEAR

8.5 **Intervention based upon implementation of clinical practice guidelines (i.e. based upon clear recommendations for practice**

Score DONE if specified in the paper;
Score NOT CLEAR if not specified in the paper;
Score NOT DONE if explicitly not based upon implementation of clinical practice guidelines.

8.6 **Clinical practice guidelines developed through formal consensus process**

Score DONE if formal consensus process described;
Score NOT CLEAR if not specified in the paper, or if intervention did not appear to be based on the implementation of clinical guidelines;
Score NOT DONE if explicitly not done.

8.7 **Recipient**

State whether each intervention was delivered to:

a) Individual

b) Group

c) NOT CLEAR

8.8 **Deliverer**

State who (or what) delivered the intervention (score all relevant):

a) Pharmacist

b) Local expert (state profession)

c) Research worker

d) Management representative

e) Computer system

f) Other (specify)

g) NOT CLEAR

8.9 **Timing**

For each intervention, state the following (for each score NOT CLEAR if information is not available):

a) Proximity to clinical decision-making (this item may be particularly relevant to audit and feedback and reminder interventions)

b) Frequency/number of intervention events

c) Duration of intervention

8.10 **Setting of intervention**

a) In practice setting

b) Not in practice setting

c) NOT CLEAR

8.11 **Source of funding**

a) Governmental organisation

b) Commercial organisation

c) Health-care provider organisation

d) Voluntary body (e.g. American Medical Association, British Medical Association)

e) Charitable trust

f) Research funding body (e.g. Medical Research Council)

g) Other (specify)

h) NOT CLEAR

8.12 **Ethical approval**

a) Score DONE if ethical approval sought and obtained for the study

b) Score NOT CLEAR if not reported

# 9. OUTCOMES

## 9.1 Description of the main outcome measure(s)

Report all the main outcomes described by the authors.

a) Health professional outcomes/process measures (e.g. the number of drugs prescribed)

b) Patient outcomes (e.g. the number of adverse drug events)

c) Economic variables

- Costs of the intervention:
  Score DONE if reported, and describe costs;
  Score NOT DONE if not reported

- Changes in direct health care costs as a result of the intervention (e.g. drugs, hospital stays etc):
  Score DONE if reported, and describe costs;
  Score NOT DONE if not reported

- Changes in non health care costs as a result of the intervention (e.g. patient travel or time off work for hospital visits):
  Score DONE if reported, and describe costs;
  Score NOT DONE if not reported

- Costs associated with the intervention are linked with provider or patient outcomes in an economic evaluation (e.g. net cost per unit change in rate of prescribing, or cost per life year saved):
  Score DONE if reported, and describe ratio;
  Score NOT CLEAR if not adequately described in the paper;
  Score NOT DONE if there was no economic evaluation reported.

## 9.2 Length of time during which outcomes were measured after initiation of the intervention

## 9.3 Length of post-intervention follow-up period

Score DONE if reported in the paper (specify length of follow-up period)
Score NOT CLEAR if not reported in the paper
Score NOT DONE if there was no follow-up period.

## 9.4 Identify a possible ceiling effect

For example, there was little room for improvement in provider performance, because it was adequate without the intervention (based on baseline measurements or control group performance).

a) Identified by investigator
- Yes
- No
- NOT CLEAR

b) Identified by reviewer
- Yes
- No
- NOT CLEAR

# 10. RESULTS

State the results as they will be entered in the review, and describe how these were calculated (e.g. relative percentage differences attributable to the intervention).

## 10.1 For RCTs and CCTs

a) State the main results of the main outcome(s), for each study group, in natural units.

b) For each available comparison, report the baseline and post intervention differences between study and control groups, in natural units. Include statistical significance if reported. Indicate if the unit of randomisation and analysis were different.

In all cases, report a more favourable provider/patient outcome in the more active intervention group as a positive (+) finding (i.e. where differences in the groups are in the intended direction).

## 10.2 For CBAs

a) State the main results of the main outcome(s), for each study group, in natural units.

b) For each study group, report baseline and post intervention results. Calculate the pre-post intervention difference for each outcome in natural units (i.e. the post-intervention outcome minus the pre-intervention outcome).

c) For each available comparison, calculate the difference across study groups of the pre-post intervention change (i.e. if, for an outcome measure $\Delta E$ is the pre-post intervention change in the experimental/intervention group, and $\Delta C$ is the pre-post intervention change in the control group, this will be $\Delta E - \Delta C$).

   Include statistical significance if reported.

   In all cases, report a more favourable provider/patient outcome in the more active intervention group as a positive (+) finding (i.e., where differences in the groups are in the intended direction).

## 10.3 For ITSs

State the main results of the main outcome(s) in natural units.

In all cases, report a more favourable provider/patient outcome attributable to the intervention as a positive (+) finding (i.e. where changes in the outcomes are in the intended direction).

a) Number of points pre and post
b) Number of patients or measurement units (eg laboratory tests) in whole series
c) Time interval between points
d) Report pre and post intervention means
e) Report absolute change in natural units
f) Report percentage relative change
g) Report the model used and statistical significance

h) ***Is information on the value of individual observations over time only reported graphically in the original paper?*** YES / NO

***In all cases, report a more favourable provider/patient outcome in the more active intervention group as a positive (+) finding (i.e., where differences in the groups are in the intended direction).***

*Notes: did you have to do any re-analysis. If yes,*

i) Report change in level and p-value in natural units

j) Report change in slope and p-value in natural units

k) Report the autocorrelation coefficient
