# Supplementary material for: Systematic review of knowledge translation strategies in the allied health professions
Source: Implement Sci. 2012 Jul 25;7:70. doi: 10.1186/1748-5908-7-70 (PMC3780719; doi:10.1186/1748-5908-7-70)
Supplement: Additional file 3 — Testing a tool for the classification of study designs in systematic reviews of interventions and exposures showed moderate reliability and low accuracy. [file 1748-5908-7-70-S3.pdf]

Journal of Clinical Epidemiology 64 (2011) 861–871

Journal of Clinical Epidemiology

Testing a tool for the classification of study designs in systematic reviews of interventions and exposures showed moderate reliability and low accuracy

Lisa Hartling[a,*], Kenneth Bond[a], P. Lina Santaguida[b], Meera Viswanathan[c], Donna M. Dryden[a]

[a]*Department of Pediatrics, Alberta Research Center for Health Evidence and the University of Alberta Evidence-based Practice Center, University of Alberta, Edmonton, Alberta, Canada T6G 2J3*

[b]*Department of Clinical Epidemiology and Biostatistics, McMaster University Evidence-Based Practice Centre, McMaster University, Hamilton, Ontario, Canada*

[c]*Division of Health Services and Social Policy Research, RTI International, Research Triangle Park, NC, USA*

Accepted 16 January 2011

Abstract

Objectives: To develop and test a study design classification tool.

Study Design: We contacted relevant organizations and individuals to identify tools used to classify study designs and ranked these using predefined criteria. The highest ranked tool was a design algorithm developed, but no longer advocated, by the Cochrane Non-Randomized Studies Methods Group; this was modified to include additional study designs and decision points. We developed a reference classification for 30 studies; 6 testers applied the tool to these studies. Interrater reliability (Fleiss' κ) and accuracy against the reference classification were assessed. The tool was further revised and retested.

Results: Initial reliability was fair among the testers ($\kappa = 0.26$) and the reference standard raters $\kappa = 0.33$). Testing after revisions showed improved reliability ($\kappa = 0.45$, moderate agreement) with improved, but still low, accuracy. The most common disagreements were whether the study design was experimental (5 of 15 studies), and whether there was a comparison of any kind (4 of 15 studies). Agreement was higher among testers who had completed graduate level training versus those who had not.

Conclusion: The moderate reliability and low accuracy may be because of lack of clarity and comprehensiveness of the tool, inadequate reporting of the studies, and variability in tester characteristics. The results may not be generalizable to all published studies, as the test studies were selected because they had posed challenges for previous reviewers with respect to their design classification. Application of such a tool should be accompanied by training, pilot testing, and context-specific decision rules. © 2011 Elsevier Inc. All rights reserved.

Keywords: Research design; Classification; Reliability; Validity; Systematic review; Observational studies; Nonrandomized studies

1. Introduction

Systematic reviews, comparative effectiveness reviews, and technology assessments aim to review and synthesize the relevant scientific literature as a basis for decision making [1]. Given the wide range of topics and outcomes addressed in this type of work, researchers frequently include nonrandomized studies to provide a detailed picture of the current knowledge and limitations of a given intervention [2–5]. Specifically, nonrandomized designs may increase the evidence base regarding long-term outcomes and safety. Nonrandomized studies may also be used to identify current limitations in evidence, recommend the types of studies that would provide stronger evidence, and guide future research [5,6].

In the context of such reviews, the appropriate classification of studies according to their design is important to guide (1) decisions around inclusion; (2) the assessment of methodological quality or risk of bias; (3) the combining

This project was funded under contract from the Agency for Healthcare Research and Quality, US Department of Health and Human Services.

The authors of this report are responsible for its content. Statements in the report should not be construed as endorsement by the Agency for Healthcare Research and Quality or the US Department of Health and Human Services.

* Corresponding author. Department of Pediatrics, Alberta Research Center for Health Evidence and the University of Alberta Evidence-based Practice Center, University of Alberta, Room 9424, 4th Floor, Aberhart Centre, 11402 University Avenue, Edmonton, Alberta, Canada T6G 2J3. Tel.: +780-492-6124; fax: +780-407-6435.

E-mail address: hartling@ualberta.ca (L. Hartling).

0895-4356/$ - see front matter © 2011 Elsevier Inc. All rights reserved.
doi: 10.1016/j.jclinepi.2011.01.010

What is new?

- We modified a tool for the classification of study designs in the context of systematic reviews, comparative effectiveness reviews, and technology assessments focusing on interventions and exposures.
- Testing showed moderate agreement ($\kappa = 0.45$) among six testers and low accuracy against a predetermined reference standard.
- We found that systematic testing and refinement enhanced the reliability of the tool.
- The classification of study designs is challenging and may be because of shortcomings of the classification tool, inadequate and inconsistent study reporting, and variation in tester characteristics.
- Application of such a tool should be accompanied by adequate training, pilot testing, and documented context-specific decision rules.
- Additional testing and refinement by other groups using different samples is encouraged and will enhance the utility of the tool.

of study results in a narrative synthesis or by statistical pooling; (4) the interpretation of findings; and (5) grading the body of evidence. Although there are a number of tools in existence to classify study designs, only one has undergone rigorous testing. Furlan et al. [7] tested a ‘‘traditional taxonomy’’ in the area of interventions for low back pain and found low reliability among reviewers despite detailed instructions and definitions. Furlan et al. [7] made a number of recommendations for future research, including developing a more comprehensive taxonomy in terms of the scope of study designs and testing the taxonomy in different fields of research.

The goal of this project was to identify a tool that could be used within the context of systematic reviews and other synthesis work focusing on interventions and exposures, and assist with the classification of study designs. The primary objectives were to (1) identify classification tools that are currently used by systematic reviewers and other researchers to classify studies according to design; (2) select the best classification tool for modification and evaluation; (3) develop instructions, including an algorithm and decision rules, for application of the modified tool to studies of interventions and exposures; and (4) test the tool and accompanying instructions for concurrent validity and interrater reliability.

2. Methods

2.1. Identification of classification tools

A sample of classification tools was compiled by contacting representatives from all the Evidence-based Practice Centers (EPCs) funded through the US Agency for Healthcare Research and Quality, other relevant organizations, and individuals with expertise in this area. Individuals were contacted by e-mail and asked to identify any taxonomies, guidelines, or other systems used to classify study designs.

2.2. Selection of classification tool for testing

The five authors ranked the tools using the following predefined criteria: (1) ease of use (i.e., contains a logic that users can readily follow); (2) unique classification for each study design (no overlap); (3) unambiguous nomenclature and decision rules/definitions (if applicable); (4) comprehensiveness in terms of range of study designs; (5) potentially allows for identification of threats to validity; and (6) developed by a well-established organization.

2.3. Development of the classification tool for testing

The selected tool was modified to incorporate relevant elements of other tools and tested through an iterative process in which the tool was applied to a sample of studies. Decisions to modify the scheme were based on the collective experience of the authors. A glossary of study design definitions and related concepts was developed to accompany the tool. The tool and glossary document were reviewed by all authors before formal testing.

While the tool was being developed, the EPCs were requested to provide examples of intervention or exposure studies, where the assignment of study design had been problematic. Two authors (L.H., K.B.), who were not involved in producing the reference classification, selected 30 sample studies from the pool of 71 studies (Appendix A). Studies were selected to cover a range of topic areas and cover all of the key decision nodes within the algorithm to ensure adequate testing of the tool. For most designs (all but two—case-control and cross-sectional) at least two sample studies were included. Additional studies were included for the designs that we felt to be more commonly encountered in systematic reviews of therapeutic interventions and exposures (e.g., before-after studies, controlled before-after studies, and cohort studies). The selection of studies was based on the design determined by two of the authors, which was not consistent in all cases with the final reference standard classification. According to the reference standard classification, all decision nodes and all but two designs (prospective cohort, nested case-control) in the algorithm were represented. A list of all 71 studies is available from the corresponding author.

2.4. *Testing and development of the reference classification*

Six individuals from the University of Alberta EPC used the tool to classify the designs of the 30 sample studies with minimal additional instruction or direction; no specific training or pilot testing was conducted (Table 1). The number of testers was based on previous work in this area [7] and published guidelines for reliability studies [8−10]. Testers were told that it would take approximately 5−10 hours to categorize the 30 studies and were asked to complete the assignment over a 2-week period. The study design names in the tool and glossary were masked, and letter codes were used in their place in an attempt to have testers work through the flow diagram in a systematic fashion rather than relying on study design labels. The tool is presented in Appendix B; the glossary is available at http://www.effectivehealthcare.ahrq.gov/index.cfm/search-for-guides-reviews-and-reports/?pageaction=displayproduct&productid=604.

Concurrently, three authors (D.D., P.L.S., M.V.) independently applied the tool to the 30 test studies to develop the reference classification, that is, the ''true'' classification for each study. Disagreements were resolved by discussion and consensus.

Overall interrater reliability was calculated using Fleiss' kappa (κ). Interrater reliability was calculated separately for the reference standard raters and the testers. As well, interrater reliability was calculated based on the level of formal training of the testers (completed relevant graduate training vs. currently enrolled in graduate training). Accuracy of the testers was measured against the reference classification and interpreted according to Landis and Koch: kappa < 0, poor agreement; 0−0.2, slight; 0.21−0.40, fair; 0.41−0.60, moderate; 0.61−0.80, substantial; 0.81−1.0, almost perfect [11]. The time taken to classify the sample of studies and the time taken per study were also recorded, and mean times were calculated.

After the first round of testing, the tool was modified further based on the results of semistructured interviews with the testers to ensure the tool's usefulness and ease of use in the context of a systematic review (Appendix C). Six testers from the University of Alberta EPC participated in a second round of testing using a random sample of 15 studies from the 30 studies used for the first round of testing. Three of the testers had been involved in the first round of testing and three had not. The three testers who were involved in the first round received no feedback after the first round of testing and were not aware of the reference standard design classifications of the sample of studies used for testing. As in the first round of testing, no specific training or pilot testing was conducted. Four of the testers received the flow diagram with the study design labels (testers in round 1 indicated a preference for unmasked labels), whereas two testers received the flow diagram with letter codes masking the study design labels. The same analyses were conducted for the second round of testing. The tool was further refined after the second round of testing (Fig. 1).

Table 1
Characteristics of testers and reference standard raters

Characteristic	Testers round 1 (n=6)	Testers round 2 (n=6)	Reference standard raters (n=3)
Education			
Undergraduate	2	2	–
Graduate, master's	4	3	–
Graduate, doctoral	–	1	3
Years of relevant experience (range)	9 mo−9 y	2 mo−9 y	4−8 y
Native language English	6	5	3

3. Results

3.1. *Identification of classification tools*

We contacted 31 organizations or individuals to identify study design classification tools. Eleven organizations or individuals responded providing 23 potential tools. All authors reviewed the tools and 10 were considered relevant for the purposes of this project (Table 2).

3.2. *Selection of classification tool for testing*

Table 2 provides the results of the ranking and observations of the tools made during the process. The three top-ranked tools were those developed by the Cochrane Non-Randomized Studies Methods Group (NRSMG), the American Dietetic Association, and the RTI International-University of North Carolina at Chapel Hill Evidence-based Practice Center (RTI-UNC EPC). The three tools were all algorithms, that is, they provided a logical sequence of ''yes or no'' decisions to make when classifying studies. None of the algorithms covered the range of study designs that systematic reviewers might encounter when conducting evidence synthesis work, particularly designs in which there is no comparator group. Further, the study nomenclature was inconsistent among the algorithms. The Design Algorithm for Studies of Health Care Interventions, developed by the Cochrane NRSMG, was considered the preferred tool and was used as the basis for further development (note that this tool is no longer advocated by the NRSMG).

3.3. *Reference classification*

The initial agreement among the three reference classification raters was fair (κ = 0.33). The three reviewers agreed on the classification of seven studies (23%), two of the three agreed on the classification of 14 (47%), and there was no agreement on the classification of nine (30%). Disagreements occurred at most decision points in the algorithm except for

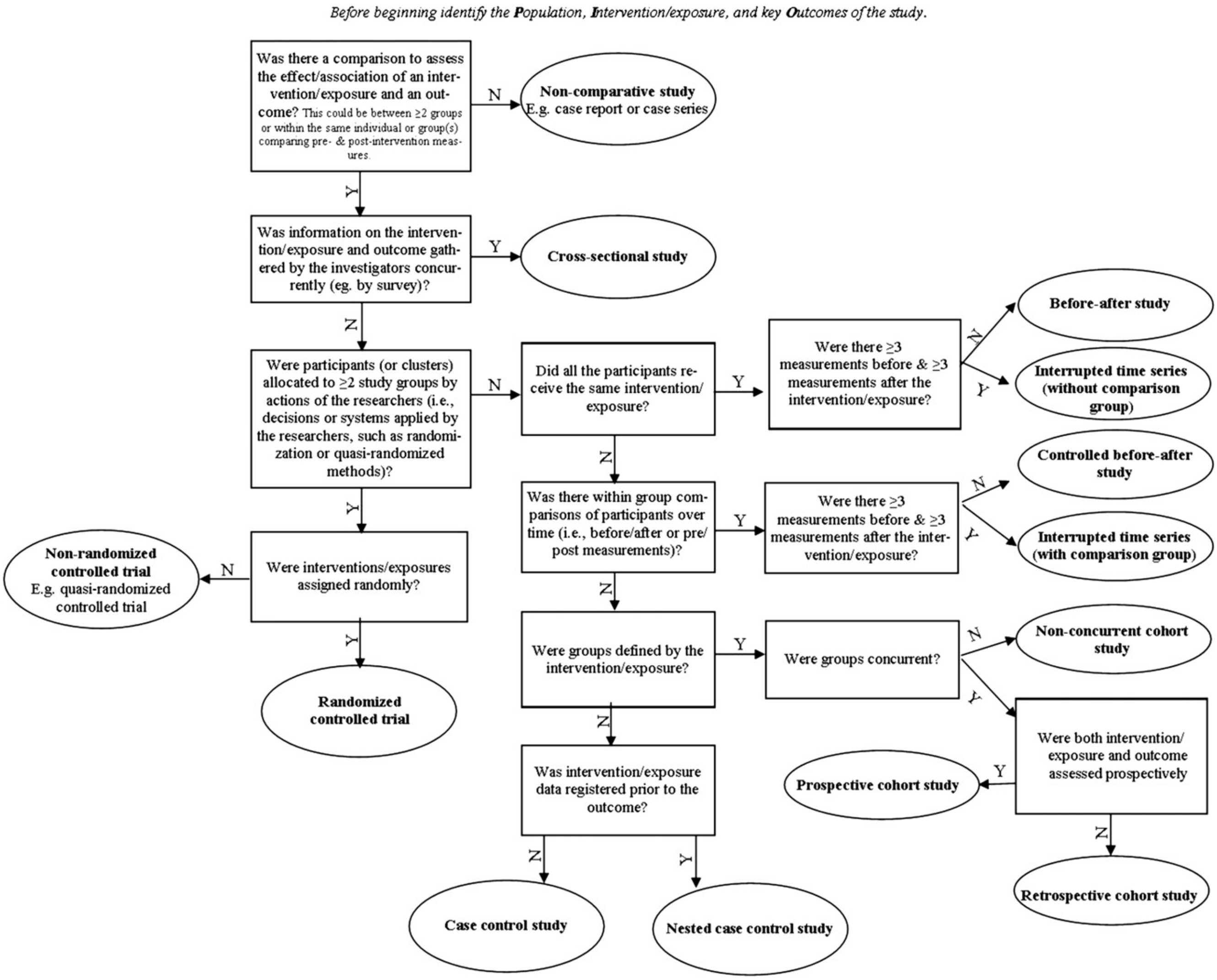

Fig. 1. Proposed design classification tool for studies of interventions and exposures.

the following three: (1) ‘‘were at least three measurements made before and after intervention/exposure?’’ (2) ‘‘was intervention/exposure data registered prior to disease?’’ and (3) ‘‘were both exposure/intervention and outcome assessed prospectively.’’ The area that created the greatest uncertainty and disagreements for the reference standard raters was the decision node ‘‘was there a single cohort?’’ Specifically, it was often difficult to determine whether the two groups under study were derived from the same cohort, and the tool did not provide any criteria to make this decision. The initial decision node (‘‘was there a comparison?’’) was also a source of disagreement. Specifically, it was unclear whether or not to classify the study as having a comparison when subgroup analyses were performed within a single group. A third point of disagreement was determining when a study was an interrupted time series (i.e., measurements taken at a minimum of three time points before and three time points after the intervention). Although there is a precedent for this definition (http://www.epoc.cochrane.org/Files/Website/Reviewer%20Resources/inttime.pdf), the number of required time points may not be universally accepted.

3.4. Phase 1 testing

3.4.1. Tester characteristics

Six testers, with varying levels of training and experience in systematic reviews, tested the modified tool. The length of time they had worked at the University of Alberta EPC ranged from 9 months to 9 years. Three of the testers had obtained a master’s degree in public health or epidemiology, and three testers were undertaking graduate level training in epidemiology or library and information sciences. The time taken to classify the 30 studies ranged from 7 to 9 hours with a mean of 8 hours overall and 16 minutes per study. Because the tool was new to the testers, this time reflects, in part, the process of familiarizing

Table 2
Study design classification tools selected for further evaluation

Tool description	Reference or source	Abbreviated tool name	Median (modal) ranking	Comments
Design algorithm for studies of health care interventions	Cochrane NRSMG[a]	DASHCI	1 (1)	Provides algorithm and definitions. Able to assign all studies from test sample into boxes.
Algorithm for classifying the research design of primary studies	ADA Evidence Analysis Manual	ADA	2 (2)	Provides algorithm and definitions. Not as easy to use as other flowcharts, not as comprehensive
Algorithm of designs for treatment studies	RTI International-University of North Carolina at Chapel Hill (UNC) EPC	RTI-UNC	3 (2)	Provides algorithm and definitions. Not comprehensive enough, not able to deal with complex designs, but clean visual lines.
Systematic literature review specification manual: study design algorithm (Appendix J)	World Cancer research fund	SLR	4 (3)	Provides algorithm and definitions. Not able to deal with complex designs, but clear nomenclature and clean visual lines.
List of study design features (Table 13.2.a) and some types of NRS designs (Box 13.1.a)	Chapter 13, written by the Cochrane NRSMG, in the Cochrane Handbook for Systematic Reviews of Interventions (Higgins & Green, 2008)[a]	Cochrane Handbook	5.5 (7)	No algorithm. Not as easy to read as a flowchart, but more comprehensive list of assignments for interventions; cannot be used to assign design, but could be used to check the response.
Traditional taxonomy of study design	Furlan 2006	Traditional	6 (4)	Provides algorithm and definitions. Not comprehensive enough, not able to deal with complex designs.
Definitions (based on Aschengrau et al. 2003; National Library of Medicine and the National Institute of Health); levels of evidence (based on Hamer and Collinson 1999)	Compiled by Minnesota EPC	Minnesota	7 (8)	No algorithm. Not clear that categories do not overlap, but some interesting additions in design (e.g., ambidirectional cohort study).
Taxonomy of quasi-experimental studies	Campbell and Stanley 1966	Campbell and Stanley	7.5 (n/a)	Provides algorithm and symbolic representation. Interesting additions to the design, but uses nomenclature that is unfamiliar which may reflect the age and/or context of the original document
Quality assessment tool for quantitative studies dictionary	McMaster University, School of Nursing, EPHPP	EPHPP	8 (9)	No algorithm. Not comprehensive enough, not able to deal with complex designs.
Research article	Brown et al. 2008	Brown	10 (10)	Not an algorithm per se, rather a description of study designs and design elements. Useful for controlled trials, does not seem as useful for cohort studies.

Abbreviations: NRSMG, Non-Randomized Studies Methods Group; EPHPP, Effective Public Health Practice Project; ADA, American Dietetic Association; NRS, nonrandomized study.

[a] These documents were produced by the same group but at different times; the most recent approach to study design classification advocated by the Cochrane NRSMG is the second tool listed which appears in the current version of the Cochrane Handbook for Systematic Reviews of Interventions available at: www.cochrane-handbook.org.

themselves with the flow diagram and the accompanying definitions.

3.4.2. Agreement

There were no studies for which all six testers agreed on the classification (Table 3). Five of six testers agreed on the classification of seven studies, four agreed on five studies, three agreed on nine studies, two agreed on eight studies. The overall level of agreement was fair ($\kappa = 0.26$). The levels of agreement for testers who had completed versus those who were undertaking graduate level training were fair ($\kappa = 0.38$) and slight ($\kappa = 0.17$), respectively.

Disagreements occurred at all decision points in the algorithm; however, testers identified the determination of a single cohort as particularly problematic. The testers also said that certain terminology in the flow diagram was unclear (e.g., ''group'' vs. ''cohort'') and that uncertainty arose because of poor study reporting. There was some variation in the manner in which testers used the flow diagram (e.g., use of glossary, working forward vs. backward through the algorithm).

3.4.3. Accuracy of testers compared with reference classification

There were no studies for which all six testers agreed with the reference classification, and there was wide variation in the testers' accuracy of classification (Table 3).

Table 3
Results of testing

Result	Phase 1 testing (30 studies)	Phase 2 testing (15 studies)
Overall agreement	κ=0.26	κ=0.45
Item agreement (number of studies)	Occurrence (%)	Occurrence (%)
6/6 testers agreed	0	3 (20)
5/6 testers agreed	7 (23)	2 (13)
4/6 testers agreed	5 (17)	6 (40)
3/6 testers agreed	9 (30)	2 (13)
2/6 testers agreed	8 (27)	2 (13)
No agreement	1 (3)	0
Number of occurrences where the specific number of testers agreed with the reference standard		
6	0	3 (20)
5	6 (20)	2 (13)
4	4 (13)	3 (20)
3	7 (23)	1 (4)
2	3 (10)	2 (13)
1	7 (23)	2 (13)
0	3 (10)	2 (13)

3.5. Phase 2 testing

3.5.1. Tester characteristics

Six staff members at the University of Alberta EPC, with varying levels of training and experience in systematic reviews, were involved in the second round of testing. Three of the testers had been involved in the first round of testing, and three of the testers had no previous involvement with the project or knowledge of the tool being tested. One tester had a PhD in Medicine, three testers had a master's degree in epidemiology, and two testers had undergraduate degrees in health sciences or related field and were undertaking graduate level training in epidemiology. The length of time the testers had worked with the University of Alberta EPC ranged from 2 months to 9 years. Four of the testers used a flow diagram that had the study design labels, whereas two of the testers used a flow diagram with letter codes. The time taken to classify the 15 studies ranged from 2.25 to 4 hours with means of 2.75 hours overall and 11 minutes per study.

3.5.2. Agreement

There were three studies for which all six testers agreed on the classification (Table 3). Five of six testers agreed on two studies, four agreed on six studies, three agreed on two studies, and two agreed on two studies. The overall level of agreement was considered moderate ($\kappa = 0.45$). The degree of agreement for testers who had completed versus those undertaking graduate level training was moderate ($\kappa = 0.45$) and fair ($\kappa = 0.39$), respectively. The difference between individuals with different levels of training was less than observed during the phase 1 testing. The level of agreement was moderate for both those who had the flow diagram with study design labels ($\kappa = 0.41$) and for those with letter codes ($\kappa = 0.55$). The agreement for testers who had and had not completed the first round of testing was fair ($\kappa = 0.36$) and moderate ($\kappa = 0.45$), respectively. For the three testers who completed both rounds of testing, intrarater reliability was fair ($\kappa = 0.33$, $\kappa = 0.34$) and moderate ($\kappa = 0.59$).

The least common agreement occurred at four key decision nodes: whether the study was an experimental design (5 of 15 studies), whether there was a comparison (4 of 15 studies), whether the assessment of exposure and outcome was prospective or retrospective (3 of 15 studies), and whether the intervention or exposure and outcome data were gathered concurrently (2 of 15 studies).

3.5.3. Accuracy of testers compared with reference classification

There were three studies for which all six testers agreed with the reference classification, but there was wide variation in the testers' accuracy of classification (Table 3).

4. Discussion

We identified over 20 tools to classify study designs and selected one for modification and testing. The final version of the modified tool showed moderate agreement among six testers and low accuracy against the predetermined reference classification. The moderate level of agreement is consistent with a previous study testing a "traditional taxonomy" [7]. The level of agreement observed in these two studies brings to light concerns about lack of agreement when using the classification tools and even greater concern with a far less transparent process when no classification tool is used at all. There are numerous tools in existence and, to our knowledge, few have undergone testing either during or after development. However, our findings also demonstrate that it is possible to systematically test and modify a tool to yield more reliable results.

There are a variety of reasons for the moderate and fair levels of agreement and accuracy observed in our study. The results likely reflect issues with the tool itself, as well as attributes of the studies that were selected for testing. The studies used during testing were identified and selected because they had posed challenges for previous reviewers with respect to their design classification. Agreement might be better with a sample of studies that is more representative of the studies that would be included in a systematic review. Further, the sample of studies that we tested covered a wide range of topics. If the studies had been on the same topic, which would be the case in a systematic review, there may have been greater reliability. One of the main reasons that the selected studies were difficult to classify was poor reporting within the studies, which resulted in the need for testers to make assumptions (e.g., whether a study was prospective or retrospective). We also found classification challenging when there were discrepancies between the intent of the investigator and the conduct of the study, between the design and how data were analyzed, and between the investigators' initial plan and their study implementation.

Moderate agreement also resulted from shortcomings of the tool itself. Many of the decision points were problematic. For example, in one-third of the studies, there were discrepancies as to whether or not the study was truly "experimental." Identifying "quasi-experimental" studies is challenging as it requires a decision about the degree of control that the investigator has over certain aspects of study design and execution. Such studies may not be considered either purely experimental (a "trial") or purely observational. This area of study design needs to be more clearly reflected in the tool. Clear guidelines are needed to interpret the extent of control that an investigator has. The practical repercussion of this uncertainty in study classification is that some "quasi-experimental" studies (e.g., before-after or controlled before-after studies) may incorrectly be classified as trials; hence, their internal validity may be exaggerated, and the results given too much weight in the context of a systematic review [12]. One design, that is, particularly problematic has been variously referred to as an "uncontrolled trial" or "single-arm trial." This design is associated with risk of bias arising from the lack of a control or comparison group. Consequently, these should be considered "before-after" studies, and our tool was designed to channel them toward this classification.

Other decision nodes that yielded inconsistent results concerned whether there was a comparison and whether the data collection was prospective or retrospective. Several factors may have contributed to this inconsistency, including a lack of clarity within the questions posed in the algorithm, the testers' relevant background knowledge, the testers' experience or training, the inconsistent use of design terminology among the studies, and lack of detailed reporting. Testers who had completed relevant graduate level training had greater agreement with the reference standard than those who were undertaking graduate level training.

We observed a fair level of agreement among the reference classification raters as well. The three reference classification raters had substantial expertise in research methods and systematic reviews: all had doctoral level training in epidemiology or research design and 4–8 years experience in systematic reviews and related research. The low level of agreement among these raters may reflect the more general complexities of study designs and the challenge of including all design considerations into a single flow diagram.

Variability in classification of studies may also reflect differences in how individuals applied or worked through the algorithm. For example, some testers worked backward or back-tracked to classify the studies according to what they felt was the most appropriate description. The testers also used the glossary accompanying the tool to varying degrees.

The difficulties in interpreting study design labels and the consequent difficulties in reaching agreement in assigning these labels to individual studies are consistent with those of other researchers. These issues have led some authors to recommend that systematic reviewers focus on features of designs rather than on design labels when assessing studies for inclusion and evaluating potential risk of bias [13]. We endorse this approach and recommend that reviewers should be as explicit as possible about the design features that are being considered. However, this does not obviate the need for or the usefulness of design labels in describing studies being considered for inclusion in systematic reviews. The use of a classification tool, including an algorithm, may provide greater transparency and consistency to the process by closely examining the design features.

4.1. Implications for practice

The appropriate classification of studies by design (or by design features) is a critical step in a systematic review to guide the selection of studies, the assessment of the risk of bias, the analysis of study results, the interpretation of results, and the grading of the body of evidence. There is a clear need for consistent use of terminology and study design labels, as well as a clear understanding of the terminology used in a particular field by those undertaking a systematic review in that field. We believe that a tool such as the one developed and tested in this study could be useful to guide this process, although the application of the tool requires several considerations to optimize agreement and reliability among reviewers.

First, training in research methods, as well as in the use of the tool, is essential. Pilot testing the tool in the context of each review is highly recommended. Second, decision rules are needed for different fields of research or review topics. Specifically, there needs to be clear decisions around how to handle a lack of clarity in study reporting. For example, when the response to a question in the algorithm is unclear, one option is to assume that the condition was not met. Documentation of the decision rules will allow for consistency and transparency. Users of the algorithm need to use standardized definitions of study designs and design features. This is what we had intended with the accompanying glossary.

4.2. Future research

The tool developed and tested in this study serves as a basis for use in systematic reviews and further research. We made minor revisions after the final round of testing that merit further testing. Research is needed to evaluate the tool within the context of a real systematic review. We encourage further testing and refinement of the tool by other groups using different samples to enhance its utility. Further research may also offer insights into the differences in reliability among individuals with varied experience, training, and education. The tool may serve as a basis for future methods work on identifying risk of bias, interpreting findings, and grading the body of evidence.

5. Conclusions

We developed and tested a tool for the classification of study designs. The level of agreement among six testers was moderate, and the accuracy against a reference classification was low. There are a number of explanations for the observed reliability and accuracy, including shortcomings of the tool, inadequate reporting of the studies, and differences in tester characteristics. Application of such a tool in the context of a systematic review should be accompanied by adequate training, pilot testing, and documented decision rules. This study demonstrates that systematic testing and refinement enhances the reliability of the tool. At the study level, clear reporting, adherence to published reporting guidelines, and appropriate and consistent use of design terminology should be enforced.

Acknowledgments

We thank Krystal Harvey for assistance with project coordination. We are very grateful to the following individuals from the University of Alberta Evidence-based Practice Center who were involved in testing the tools and providing feedback: Ahmed Abou-Setta, Liza Bialy, Michele Hamm, Nicola Hooton, David Jones, Andrea Milne, Kelly Russell, Jennifer Seida, and Kai-On Wong. We thank Ben Vandermeer for conducting the analyses. We thank Karen Lohman Siegel from the Agency for Healthcare Research and Quality for her input and advice during the course of the project. We thank Stephanie Chang from the Agency for Healthcare Research and Quality for critical review of the final report. We are also appreciative of the individuals who provided sample classification tools and of the members of other Evidence-based Practice Centers who provided the studies that were used for testing.

Appendix A

Studies used for testing

[Details of individual studies available at http://www.effectivehealthcare.ahrq.gov/index.cfm/search-for-guides-reviewss-and-reports/?pageaction=displayproduct&productid=604]

Anderson K, Boothby M, Aschenbrener D, van Holsbeeck Marnix. Outcome and structural integrity after arthroscopic rotator cuff repair using 2 rows of fixation. Am J Sport Med. 2006; 34(14):1899–905.

Bentas W, Wolfram M, Jones J, Bräutigam R, Kramer W, Binder J. Robotic technology and the translation of open radical prostatectomy to laparoscopy: The early Frankfurt experience with robotic radical prostatectomy and one year follow-up. Eur Urol. 2003; 44: 175–81.

Blais L, Couture J, Rahme E, LeLorier J. Impact of a cost sharing drug insurance plan on drug utilization among individuals receiving social assistance. Health Policy. 2003; 64:163–72.

Boszotta H, Prünner K. Arthroscopically assisted rotator cuff repair. Arthroscopy. 2004; 20(6):620–6.

Cardo DM, Culver DH, Ciesielski CA, Srivastava PU, Marcus R, Abiteboul D, et al. A case-control study of HIV seroconversion in health care workers after percutaneous exposure. The N Engl J Med. 1997; 337(21):1485–90.

Carey TS, Evans A, Hadler N, Kalsbeek W, McLaughlin C, Fryer J. Care-seeking among individuals with chronic low back pain. Spine. 1995; 20(3):312–7.
Chenot J-F, Becker A, Leonhardt C, Keller S, Donner-Banzhoff N, Baum E, et al. Determinants for receiving acupuncture for LBP and associated treatments: a prospective cohort study. BMC Health Serv Res. 2006; 6(149).
Cherkin DC, Deyo RA, Sherman KJ, Hart LG, Street JH, Hrbek A, et al. Characteristics of visits to licensed acupuncturists, chiropractors, massage therapists, and naturopathic physicians. J Am Board Fam Pract. 2002; 15(6):463–72.
Cranson RW, Orme-Johnson DW, Gackenbach J, Dillbeck MC, Jones CH, Alexander CN. Transcendental meditation and improved performance on intelligence-related measures: Longitudinal study. Pers Individ Dif. 1991; 12(10):1105–16.
Darai E, Jeffry L, Deval B, Birsan A, Kadoch O, Soriano D. Results of tension-free vaginal tape in patients with or without vaginal hysterectomy. Eur J Obstet Gynecol Reprod Biol. 2002; 103:163–7.
Davies HTO, Crombie IK, MacRae WA, Rogers KM, Charlton JE. Audit in pain clinics, Changing the management of low-back and nerve-damage pain. Anesthesia. 1996; 51:641–6.
DeVader SR, Neeley HL, Myles TD, Leet TL. Evaluation of gestational weight gain guidelines for women with normal prepregnancy body mass index. Obstet Gynecol. 2007; 110(4):745–51.
Happ MB, Sereika S, Garrett K, Tate J. Use of the quasi-experimental sequential cohort design in the Study of Patient-Nurse Effectiveness with Assisted Communication Strategies (SPEACS). Contemp Clin Trials. 2008; 29:801–8.
Harris CM, Scrivener G. Fundholders' prescribing costs: the first five years. BMJ. 1996; 313:1531–4.
Herman RM, Richter P, Walega P, Popiela T. Anorectal sphincter function and rectal barostat study in patients following transanal endoscopic microsurgery. Int J Colorectal Dis. 2001; 16:370–6.
Hollabaugh RS, Dmochowski RR, Kneib TG, Steiner MS. Preservation of putative continence nerves during radical retropubic prostatectomy leads to more rapid return of urinary continence. Urology. 1998; 51:960–7.
Kaplan SA, Santarosa RP, Te AE. Comparison of fascial and vaginal wall slings in the management of intrinsic sphincter deficiency. Urology. 1996; 47:885–9.
Karlsson I, Bondemark L. Intraoral Maxillary Molar Distalization, Movement before and after Eruption of Second Molars. Angle Orthod. 2006; 76(6): 923–9.
Leclercq C, Victor F, Alonso C, Pavin D, Revault d'Allones G, Bansard JY, et al. Comparative effects of permanent biventricular pacing for refractory heart failure in patients with stable sinus rhythm or chronic atrial fibrillation. Am J Cardiol. 2000; 85:1154–6.
Lipscomb HJ, Li L, Dement J. Work-related falls among union carpenters in Washington State before and after the vertical fall arrest standard. Am J Ind Med. 2003; 44:157–65.
Minassian VA, Al-Badr A, Pascali DU, Lovatsis D, Drutz HP. Tension-Free Vaginal Tape: Do patients who fail to follow-up have the same results as those who do? Neurourol Urodyn. 2005; 24:35–8.
Paulson DL, Wash L. A comparison of wait times and patients leaving without being seen when licensed nurses versus unlicensed assistive personnel perform triage. J Emerg Nurs. 2004; 30(4):307–11.
Qin L, Au S, Choy W, Leung P, Neff M, Lee K, et al. Regular Tai Chi Chuan exercise may retard bone loss in postmenopausal women: A Case-Control Study. Arch Phys Med Rehabil. 2002; 83:1355–9.
Scheurmier N, Breen AC. A pilot study of the purchase of manipulation services for acute low back pain in the United Kingdom. J Manipulative Physiol Ther. 2009; 21(1):14–8.
Sit JWH, Yip VYB, Ko SKK, Gun APC, Lee JSH. A quasi-experimental study on a community-based stroke prevention programme for clients with minor stroke. J Clin Nurs. 2007; 16:272–81.
Verrotti A, Chiarelli F, Sabatino G, Blasetti A, Tumini S, Morgese G. Education, knowledge and metabolic control in children with type 1 diabetes. Eur Rev Med Pharmacol Sci. 1993; 15:5–10.
Wells NM, Yang Y. Neighborhood design and walking. Am J Prev Med. 2008; 34(4):313–9.
Wickizer TM, Kopjar B, Franklin G, Joesch J. Do drug-free workplace programs prevent occupational injuries? Evidence from Washington State. Health Serv Res. 2004; 39(1):91–110.
Wilson TE, Fraser-White M, Feldman J, Homel P, Wright S, King G, et al. Hair salon stylists as breast cancer prevention lay health advisors for African American and Afro-Caribbean women. J Health Care Poor Underserved. 2008; 19:216–26.
Zanconato S, Baraldi E, Santuz P, Magagnin G, Zacchello F. Effect of inhaled disodium cromoglycate and albuterol on energy cost of running in asthmatic children. Pediatr Pulmonol. 1990; 8:240–4.

Appendix B

Classification tool used for first round of testing

Study design labels were masked for testing. See Fig. 1 and Appendix C for study designs.

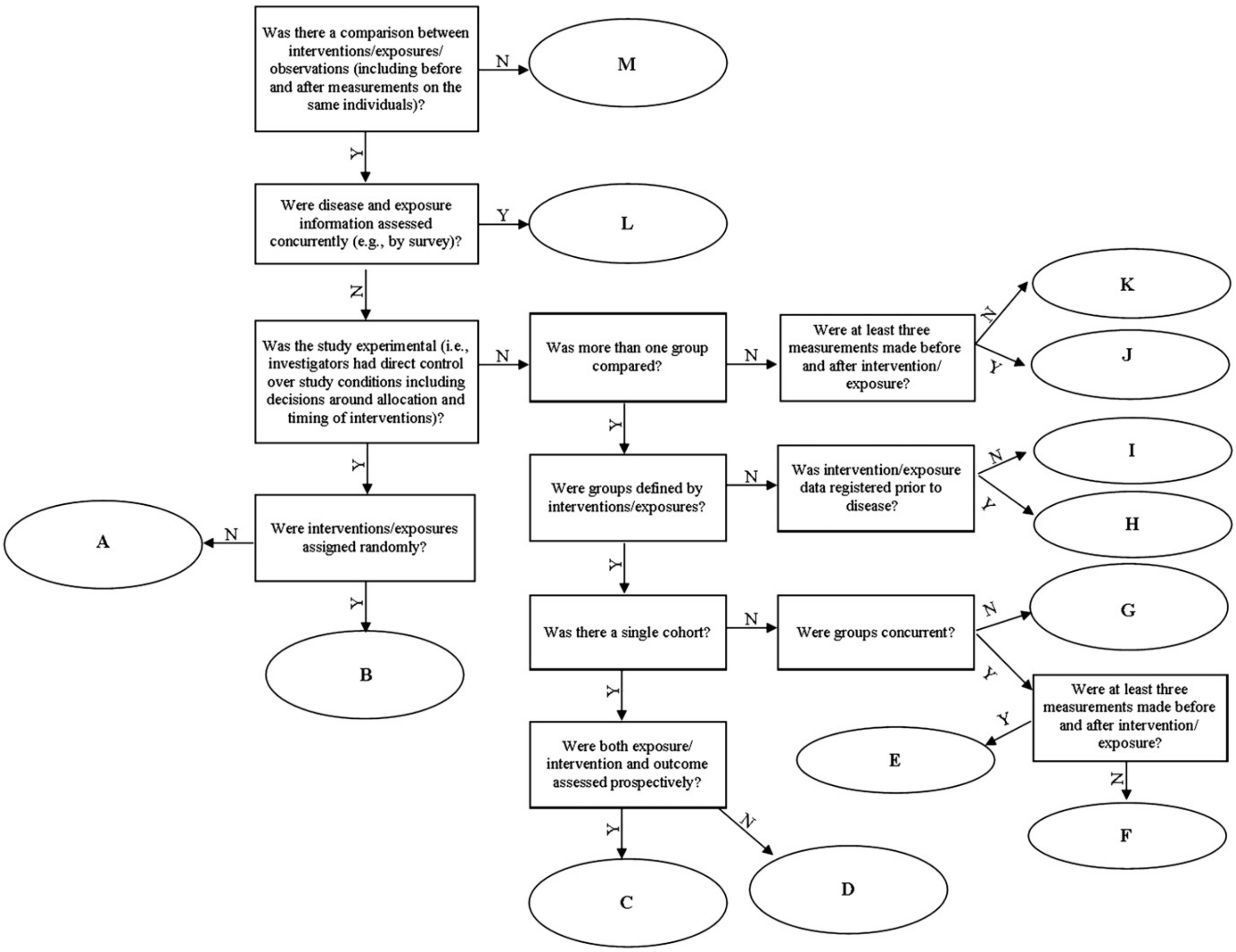

Appendix C

Classification tool used for second round of testing

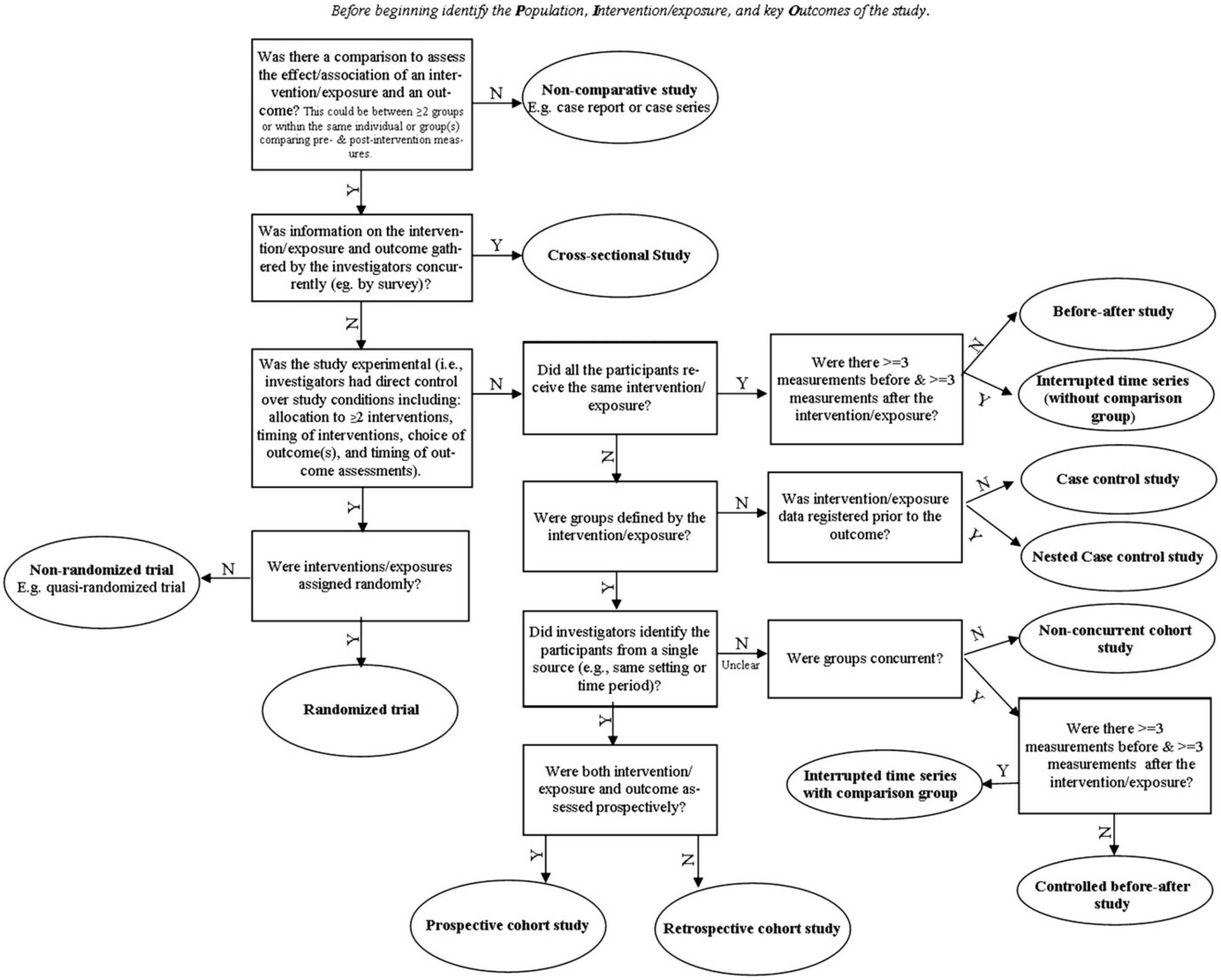

References

[1] Agency for healthcare research and quality. Evidence-based practice centres [webpage]. Available at http://www.ahrq.gov/clinic/epc/. Accessed October 2008.
[2] Egger M, Davey Smith G, Schneider M. Systematic reviews of observational studies. In: Egger M, Davey Smith G, Altman DG, editors. Systematic reviews in health care: meta-analysis in context. London, UK: BMJ; 2001. p. 211–27.
[3] Chou R, Helfand M. Challenges in systematic reviews that assess treatment harms. Ann Intern Med 2005;142:1090–9.
[4] McCulloch P, Taylor I, Sasako M, Lovett M, Griffin D. Randomised trials in surgery: problems and possible solutions. BMJ 2002;324:1448–51.
[5] Reed D, Price EG, Windish DM, Wright SM, Gozu A, Hsu EB, et al. Challenges in systematic reviews of education intervention studies. Ann Intern Med 2005;142:1080–9.
[6] Atkins D, Fink K, Slutsky J. Better information for better health care: the evidence-based practice center program and the agency for healthcare research and quality. Ann Intern Med 2005;142:1035–41.
[7] Furlan AD. Non-randomized studies: an evaluation of search strategies, taxonomy and comparative effectiveness with randomized trials in the field of low-back pain [dissertation]. University of Toronto; 2006.
[8] Walter SD, Eliasziw M, Donner A. Sample size and optimal study designs for reliability studies. Stat Med 1998;17:101–10.
[9] Donner A, Eliasziw M. Sample size requirements for reliability studies. Stat Med 1987;6:441–8.
[10] Eliasziw M, Young SL, Woodbury MG, Fryday-Field K. Statistical methodology for the concurrent assessment of interrater and intrarater reliability: using goniometric measurements as an example. Phys Ther 1994;74:788.
[11] Landis JR, Koch GG. The measurement of observer agreement for categorical data. Biometrics 1977;33:159–74.
[12] Ioannidis JPA, Haidich A-B, Pappa M, Pantazis N, Kokori SI, Tektonidou MG, et al. Comparison of evidence of treatment effects in randomized and nonrandomized studies. JAMA 2001;286:821–30.
[13] Higgins JPT, Green S. Cochrane handbook for systematic reviews of interventions 5.0.1 [September 2008]. Chichester, UK: John Wiley & Sons, Ltd; 2008.
