# Supplementary material for: Systematic review of knowledge translation strategies in the allied health professions
Source: Implement Sci. 2012 Jul 25;7:70. doi: 10.1186/1748-5908-7-70 (PMC3780719; doi:10.1186/1748-5908-7-70)
Supplement: Additional file 4 — WIDER Recommendations to Improve Reporting of the Content of Behaviour Change Interventions. [file 1748-5908-7-70-S4.pdf]

WIDER Recommendations to Improve Reporting of the Content of Behaviour Change Interventions

The following four recommendations to journal editors are made on behalf of the **W**orkgroup for **I**ntervention **D**evelopment and **E**valuation **R**esearch (WIDER) group and colleagues supporting WIDER's aims. WIDER's overarching aim is to improve the scientific reporting of behavior change interventions (BCIs) and evaluations of BCIs in accordance with CONSORT statements. We believe that CONSORT guidance and APA reporting standards (APA, 2008) need to be extended to allow better communication of the content of BCIs and active controls. The information specified by recommendations 1-3 is essential if BCIs are to be understood, replicated, accurately portrayed in literature reviews (including meta analyses) and contribute to improved professional practice. The information specified in recommendation 4 is critical to understanding what we mean by an "effective" intervention. The changes to editorial policy we recommend could greatly accelerate progress in the science of behaviour change and maximise the impact of such scientific endeavour on applications, including health promotion and patient care. Our recommendations focus on BCIs and BCI evaluations but these recommendations may be relevant to a broader range of psychological interventions designed to change cognitions, emotions and physiological responses.

1. Detailed Description of Interventions in Published Papers

Current standards of reporting of behaviour change intervention (BCI) evaluations mean that researchers and practitioners often cannot fully understand and replicate interventions evaluated in the literature. We suggest that journal editors adopt the guidance provided by the extended CONSORT statements for reporting of trials of Nonpharmacologic Treatments (Boutron, Moher, Altman, Schulz, & Ravaud, 2008) and pragmatic trials (Zwarenstein et al., in press). These are usefully augmented for behavioural scientists by Davidson et al (2003).

In particular, *we recommend* that instructions to authors specify that BCI evaluations describe (1) characteristics of those delivering the intervention, (2) characteristics of the recipients (and see Noguchi et al., 2007, for unusual but importantly informative detail on participants before and after attrition) (3) the setting (e.g., worksite, time and place of intervention), (4) the mode of delivery (e.g., face-to-face) (5) the intensity (e.g., contact time), (6) the duration (e.g., number of sessions and their spacing over a given period), (7) adherence/ fidelity to delivery protocols and (8) a detailed description of the intervention content provided for each study group.

2. Clarification of Assumed Change Process and Design Principles

Readers and reviewers need to be aware of, and have access to, the principles that underpin researchers' development of BCIs because this facilitates replication and adoption of the findings. For example, what change processes were thought to be necessary to prompt a change in the specified behaviour? How was the intervention design informed by theoretical considerations, if at all? What change techniques were incorporated in the intervention and why? Examples, of papers clarifying these design issues include Araújo-Soares et al. (in press) and Van Kesteren, Kok, Hospers, Schippers & De Wildt (2006).

Consequently, *we recommend* that instructions to authors specify that BCI evaluations describe (1) the intervention development, (2) the change techniques used in the intervention, and (3) the causal processes targeted by these change techniques; all in as

much detail as is possible, unless these details are already readily available (e.g., in a prior publication).

3. Access to Intervention Manuals/ Protocols

As CONSORT specifies, reviewers, researchers and practitioners also need to know what exactly was done in each intervention, that is, what materials were used in what sequence etc. For most BCIs, this material is usually too detailed to be included in a published intervention report. Unfortunately, however, protocols or manuals describing interventions are often not available from authors after BCI evaluations are published. Indeed, a few years after publication, it may be impossible to discover exactly what was tested in a BCI evaluation.

Consequently, *we recommend* that, at the time of publishing a BCI evaluation report, editors ask authors submit protocols or manuals describing BCI evaluations or, alternatively, specify where manuals can be easily and reliably accessed by readers. Such supplementary materials can be made accessible online.

Note that recommendation 3 has already been adopted as policy by some journals (e.g., West, 2008). Note too that in special cases limited access to online manuals can be provided (e.g., to comply with copyright demands) and that authors should be allowed to submit manuals and supplementary intervention materials in their own language (so that translations costs do not prohibit publication). In future we hope that that manuals and protocols would be available prior to evaluation studies being undertaken and also made available to reviewers of BCI evaluation reports so that they can better evaluate the aims, method, operationalisation and fidelity of intervention delivery. Finally, supplementary materials could also include video recordings of intervention techniques (e.g., classes and demonstrations). A precedent has already been set for such reporting by e.g., the *Journal of Visualized Experiments*.

4. Detailed Description of Active Control Conditions

Observed BCI efficacy/effectiveness is relative either to a baseline measure of behaviour, an active control group, or both. In trials that include control groups efficacy/effectiveness depends in part on the impact of the techniques employed in the control condition. For example, an intervention that is effective when compared to poor routine care may not improve on state-of-the-art routine care. If reviewers and researchers do not know the content of active control conditions and what effect these conditions are likely to have, they may misinterpret intervention quality and efficacy/ effectiveness data. Boutron et al. (2008) note, for example, that, “if the control treatment is usual care, authors should report all the components received by the control group” but that this is rarely achieved in practice p.288).

Consequently, *we recommend* that instructions to authors specify that BCI evaluations describe the content of active control groups in as much detail as is possible (e.g., the techniques used) in a similar manner to the description of the content of the intervention Itself. Consistent with point 2 (above), as standardised terminology is developed for this purpose, better descriptions of active control groups will evolve over time but much could be done to improve current practice.

References

APA Publications and Communications Board Working Group on Journal Article Reporting Standards (2008). Reporting Standards for Research in Psychology Why Do We Need Them? What Might They Be? *American Psychologist, 63,* 839-851.

Araújo-Soares, V., McIntyre, T., MacLennan, G., Sniehotta, F., F. (in press) Development and exploratory cluster-randomised opportunistic trial of theory-based intervention to enhance physical activity among adolescents. *Psychology & Health.*

Boutron, I., Moher, D., Altman, D. G., Sc, Schulz, K. F. & Ravaud, P. for the CONSORT Group (2008). Extending the CONSORT Statement to Randomized Trials of Nonpharmacologic Treatment: Explanation and Elaboration. *Annals of Internal Medicine, 148*, 295-309.

Davidson, K., W., Goldstein, M., Kaplan, R. M., Kaufmann, P., G., Knatterund, G., L., Orleans, C., T., Spring, B., Trudeau, K., J. & Whitlock, E. P. (2003). Evidence-based behavioral medicine: What is it and how do we achieve it? *Annals of Behavioral Medicine, 26*, 161-171.

Noguchi, K., Albarracín, D., Durantini, M R. & Glasman, L. R. (2007). Who participates in which health promotion programs? A meta-analysis of motivations underlying enrolment and retention in HIV-prevention interventions. *Psychological Bulletin*, *133*, 955 975.

Van Kesteren, N., C., N, Kok, G., Hospers, H., Schippers, J. & De Wildt, W. (2006). Systematic development of a self-help and motivational enhancement intervention to promote sexual health in HIV-Positive men who have sex with men. *AIDS Patient Care and STDs, 20.* 858-875.

West, R. (2008). Providing full manuals and intervention descriptions: Addiction policy. *Addiction, 103*, 1411.

Zwarenstein, M., Treweek, S., Gagnier, J. J., Altman, D. G., Tunis, S., Haynes, B., Oxman, A., & Moher, D. (in press). Improving the reporting of pragmatic trials: an extension of the CONSORT Statement. *British Medical Journal.*

Written and/or endorsed by

Professor Charles Abraham, University of Sussex, UK
and *Chair of WIDER*
Professor Dolores Albarracín, University of Illinois, USA.
Dr. Vera Araújo-Soares, Robert Gordon University, UK.
Dr. L. Kay Bartholomew, University of Texas, USA.
Professor Paul Bennett, University of Cardiff, UK
and editor of the *British Journal of Health Psychology*.
Professor Michael C. Costanza, Rushden, Northamptonshire UK
and *Statistical editor of Preventive Medicine*.
Professor David Crawford. Deakin University, Australia
and *Editor-in-Chief of the International Journal of Behavioral Nutrition and Physical Activity*
Professor Karina Davidson, Columbia University College of Physicians and Surgeons, USA and *Associate Editor of Health Psychology.*
Mr. Marijn de Bruin, University of Wageningen, The Netherlands.
Professor Nanne de Vries, University of Maastricht, The Netherlands.
Professor Martin Eccles Institute of Health and Society, Newcastle University
and *Co-Editor in Chief of Implementation Science.*
Professor Robbie Foy, Leeds Institute of Health Sciences, University of Leeds
and *Associate Editor of Implementation Science.*
Professor Gaston Godin, Université Laval, Canada.
Professor Robert Kaplan, Wasserman Distinguished Professor, UCLA, USA
and *Editor of Health Psychology.*
Professor Gerjo Kok, University of Maastricht.
Professor Blair Johnson, University of Connecticut, USA.
Professor Marie Johnston, University of Aberdeen, UK.
Professor Susan Michie, University College London.
Professor Rona Moss-Morris, University of Southampton, UK
and *Editor of Psychology & Health.*
Dr. Melissa Norton, BioMed Central LTD, Middlesex House, London
and *Editorial Director (Medicine),* BioMed Central
Dr. Jigisha Patel, BioMed Central LTD, Middlesex House, London
and Medical Editor of *BMC Public Health*
Dr. Gjalt-Jorn Peters, University of Maastricht, The Netherlands.
Dr. Rob Ruiter, University of Maastricht, The Netherlands
and *Associate Editor of Psychology & Health.*
Professor Herman Schaalma, University of Maastricht, The Netherlands
Professor Paschal Sheeran, University of Sheffield, UK.
Professor Lorraine Sherr, University College London.
Editor of AIDS Care, Psychology Health and Medicine
and *Editor of Vulnerable Children and Youth Studies*.
Dr. Falko Sniehotta, University of Aberdeen, UK.
Dr. Shaun Treweek, University of Dundee, UK.
Professor Joop van der Pligt, University of Amsterdam,The Netherlands
and *Editor of Health Psychology Review.*
Dr. Kavita Vedhara, University of Bristol, UK
and *Editor of the British Journal of Health Psychology.*
Professor Robert West, University College London, UK
and *Editor of Addiction.*
Professor Lucy Yardley, University of Southampton, UK
and *Editor of Psychology & Health*.
