# Supplementary material for: Systematic review of knowledge translation strategies in the allied health professions
Source: Implement Sci. 2012 Jul 25;7:70. doi: 10.1186/1748-5908-7-70 (PMC3780719; doi:10.1186/1748-5908-7-70)
Supplement: Additional file 6 — Quality assessment tool for qualitative studies. [file 1748-5908-7-70-S6.doc]

**Additional File 3 – *Quality Assessment Tool for Qualitative Studies***
