# Supplementary material for: Systematic review of knowledge translation strategies in the allied health professions
Source: Implement Sci. 2012 Jul 25;7:70. doi: 10.1186/1748-5908-7-70 (PMC3780719; doi:10.1186/1748-5908-7-70)
Supplement: Additional file 7 — Assessment of 29 quantitative studies and three qualitative studies using separate tools. [file 1748-5908-7-70-S7.pdf]

Additional file 7: *Assessment of 29 quantitative studies and three qualitative studies using separate tools*

Author (Year)	*Quality Assessment Tool for Quantitative Studies* rating
**Quantitative Studies (n=29)**	
None	**Strong**
Bekkering, GE, van Tulder MW, et al. (2005)	**Moderate**
Dualde, E, et al. (2009)	
Egen, V, et al. (2003)	
Hoffmann, W, et al. (2008)	
Stevenson, K, et al. (2006)	
Tripicchio, B, et al. (2009)	
Airkasinen, M, et al. (1998)	**Weak**
Banz, M, et al. (2004)	
Bekkering, GE, Hendricks HJM, et al. (2005)	
Benrimoj, SI, et al. (2007)	
Bracchi, RCG, et al. (2005)	
Brooks, VG, et al. (2001)	
Brown, CJ et al. (2005)	
Brug, J, et al. (2007)	
Fjortoft, N, et al. (2003)	
Fjortoft, N, et al. (2007)	
Gross, DP, et al. (2009)	
Hammond, A, et al. (2005)	
Hirsch, JD, et al. (2009)	
Hoeijenbos, M, et al. (2005)	
Johnson, ST, et al. (2007)	
Kerssens, JJ, et al. (1999)	
Martin, BA, et al. (2010)	
McCluskey, A, et al. (2005)	
McKenna, K, et al. (2005)	
Munroe, WP, et al. (1997)	
Nikopoulou-Smyrni, P, et al. (2007)	
Pennington, L, et al. (2005)	
Rebbeck, T, et al. (2006)	

Quality Assessment Tool for Qualitative Studies rating	Author (Year)
**Qualitative Studies (n=3)**	
**5**	Vachon, B, et al. (2009)
**4**	None
**3**	None
**2**	Schreiber, J, et al. (2009)
**1**	Molfenter, SM, et al. (2009)
